# *Track-A-Worm*, An Open-Source System for Quantitative Assessment of *C. elegans* Locomotory and Bending Behavior

**DOI:** 10.1371/journal.pone.0069653

**Published:** 2013-07-26

**Authors:** Sijie Jason Wang, Zhao-Wen Wang

**Affiliations:** 1 University of Connecticut Health Center, Farmington, Connecticut, United States of America; 2 Department of Neuroscience, University of Connecticut Health Center, Farmington, Connecticut, United States of America; Imperial College London, United Kingdom

## Abstract

A major challenge of neuroscience is to understand the circuit and gene bases of behavior. *C. elegans* is commonly used as a model system to investigate how various gene products function at specific tissue, cellular, and synaptic foci to produce complicated locomotory and bending behavior. The investigation generally requires quantitative behavioral analyses using an automated single-worm tracker, which constantly records and analyzes the position and body shape of a freely moving worm at a high magnification. Many single-worm trackers have been developed to meet lab-specific needs, but none has been widely implemented for various reasons, such as hardware difficult to assemble, and software lacking sufficient functionality, having closed source code, or using a programming language that is not broadly accessible. The lack of a versatile system convenient for wide implementation makes data comparisons difficult and compels other labs to develop new worm trackers. Here we describe *Track-A-Worm*, a system rich in functionality, open in source code, and easy to use. The system includes plug-and-play hardware (a stereomicroscope, a digital camera and a motorized stage), custom software written to run with Matlab in Windows 7, and a detailed user manual. Grayscale images are automatically converted to binary images followed by head identification and placement of 13 markers along a deduced spline. The software can extract and quantify a variety of parameters, including distance traveled, average speed, distance/time/speed of forward and backward locomotion, frequency and amplitude of dominant bends, overall bending activities measured as root mean square, and sum of all bends. It also plots worm travel path, bend trace, and bend frequency spectrum. All functionality is performed through graphical user interfaces and data is exported to clearly-annotated and documented Excel files. These features make *Track-A-Worm* a good candidate for implementation in other labs.

## Introduction

The nematode *Caenorhabditis elegans* moves in a sinusoidal fashion on the surface of agar plates under laboratory conditions. This locomotion behavior relies on muscle contractions, neuromuscular transmission, and a neural circuit consisting of a small number of locomotory control neurons and motor neurons. It adapts to particular environments of the worm through sensory inputs. Mutations of many genes may cause changes in *C. elegans* locomotion behavior. Therefore, detection and characterization of abnormal locomotory behavior in mutant worms are of tremendous value to delineating functional neural circuits, identifying cellular signaling pathways, and interpreting gene functions. Traditionally, mutants with abnormal behavior were visually identified and loosely described as uncoordinated, sluggish, loopy, kinky, jerky, and so on. This approach was highly subjective and often not sufficiently sensitive. In recent years, a number of computer-based worm tracking systems have been developed for quantitative analyses of *C. elegans* locomotion behavior. These systems can be divided into two classes: single-worm trackers and multi-worm trackers. Generally, single-worm trackers may extract detailed phenotypic features from worms at a high magnification [1]–[5] whereas multi-worm trackers are limited to extracting movement metrics such as speed and travel path from worms at a low magnification [6]–[10]. These automated worm tracking systems have greatly improved ways of detecting and characterizing mutant behavior.

A multi-worm tracker developed by the Goodman lab [8] has been used by many other labs. However, there has not been a single-worm tracker with wide implementations. The Sternberg lab and the Schafer lab were among the first developing feature-rich single-worm trackers [1], [2]. The hardware designs and software, including subsequent updates, have been available to the public (http://wormlab.caltech.edu/publications/download.html; http://www.mrc-lmb.cam.ac.uk/wormtracker/). However, only a few other labs appear to have implemented either of the two systems based on a search of publications using Textpresso for *C. elegans* (http://www.textpresso.org/celegans/) [11]–[14]. Meanwhile, other labs continued to develop new systems, which were often not as feature-rich as the two earlier systems although, in some cases, allowed optogenetic stimulation or calcium imaging [3]–[5], [15]–[22]. The lack of a widely accepted single-worm tracker is for several common reasons, including hardware that is difficult to assemble, software that lacks sufficient functionality or graphical user interface and requires a specially trained person to run, closed software source code that prevents custom modifications, use of a programming language that is not broadly accessible, data output that is difficult to interpret, and the absence of a detailed user manual. The constant development of new tracking systems not only wastes resources, but also makes the comparison of data from different labs difficult. The *C. elegans* research community could potentially benefit from an automated single-worm tracker with that is rich in functionality, easy to implement, and convenient to add new features. *Track-A-Worm* was developed in the hope to serve such a purpose.

*Track-A-Worm* has several key features. First, *Track-A-Worm* has rich functionality. It can provide detailed quantification of worm locomotion and bending properties, including the use of root-mean-square (RMS) as an effective measure for small or chaotic oscillations, which is a feature not found in other worm trackers. Second, *Track-A-Worm* is a plug-and-play system based on commercially available hardware and Windows operating systems. Third, *Track-A-Worm* is easy to use with all functionality performed through graphical user interfaces. Fourth, *Track-A-Worm* is easy for end users to add new features because it was built on the basis of the powerful and versatile Matlab, and has open source code.

*Track-A-Worm* has been successfully used to detect and quantify changes in locomotion speed and head bending properties of several mutants, including loss-of-function mutants of *slo-1*/BK channel, *dyb-1*/dystrobrevin, *ctn-1*/α-catulin, and *bkip-1*/BK channel interacting protein-1 [23]–[25]. Its usefulness is further supported by the comparison of forward/backward locomotion and body bending properties between wild-type worms and an *unc-9/innexin* mutant, which were chosen because the mutants display complex abnormalities in locomotion behavior (**File S1**). A detailed user manual is provided in **File S2**. The software is available for download at http://zwwang.uchc.edu/wormtrack/index.html.

## Methods

### Hardware

The hardware components of *Track-A-Worm* include a stereomicroscope, a *xy* motorized stage, a digital camera, and a Windows 7 (32-bit) computer. The computer is installed with Matlab R2012b with several required tool boxes, including Image Processing Toolbox, Image Acquisition Toolbox, Instrument Control Toolbox, and Signal Processing Toolbox. Matlab drivers for the camera and stage are designed to be modular, so only a few functions must be replaced to accommodate different hardware. When choosing alternative hardware, please note the following: (1) choose a fast computer with connecting ports for the camera and motorized stage; (2) choose a stage with *xy* travel distances longer than the diameter of the petri dish used in worm tracking; and (3) choose a camera that has driver support within Matlab. A large monitor with a high resolution (*e. g.* 23 or 24 inch, 1920×1080) is highly recommended. Compatibility with 64-bit versions of Matlab and Windows operating systems has not been tested, although theoretically functionality should be preserved assuming the availability of 64-bit system hardware drivers. The specific hardware components of our system are described in the user manual (**File S2**) and listed at the software download website (http://zwwang.uchc.edu/wormtrack/index.html). Most components, except for the motorized stage, are commonly used in *C. elegans* research labs. Thus, a typical worm lab may only have to purchase one or two new items to set up the system.

### Tracking and Analysis Algorithm

To describe worm locomotion and body shape, there are several important issues to address. First, a moving worm needs to be maintained in the camera imaging field at a sufficient magnification. Second, the worm outline and head direction need to be determined. Third, a spline representing the midline of the worm at each frame needs to be deduced. Finally, parameters related to worm locomotion and body shape need to be quantified.

#### Stage tracking

A motorized stage is used to keep a crawling worm centered in the camera imaging field. *Track-A-Worm* compares positions of the worm over successive images, and uses this information to correct the stage position at 1-sec intervals. All stage movements are automatically recorded in a *stage* file, which is used in combination with worm image files in subsequent analyses. Stage positions are corrected between intervals of image acquisitions. Thus there are neither blurred images nor discontinuities in the reconstructed worm path due to stage movements.

#### Image processing

**Figure 1:**
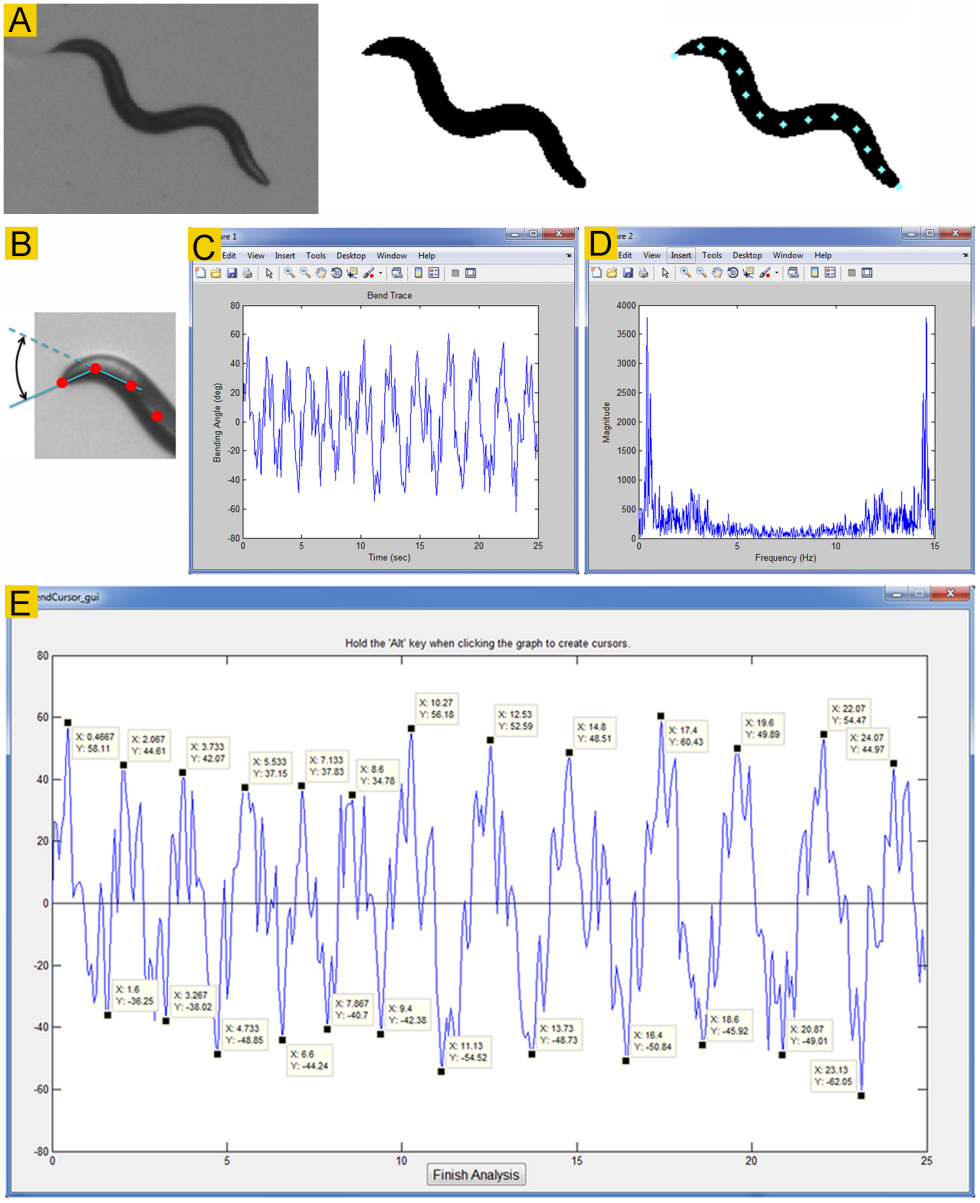
Image binarization and bending activity analyses. **A**. *Track-A-Worm* converts a gray worm image (*left*) into a binary image (*middle*), identifies the head (indicated by “x”), and places 13 markers along a deduced spline at equal intervals (*right*). **B**. Diagram of the first bend, which is the complementary angle formed by the two straight lines between markers #1 (the nose) and #2, and between markers #2 and #3. **C**. Bend trace of the first bend of a wild-type worm. **D**. Bend frequency spectrum generated by Fourier transformation of the bend trace, which appears as a mirror image. The user should disregard the second half of the graph. The main peak of this graph indicates that the dominant bending frequency is ∼0.4 Hz. **E**. Quantification of the maximum bend from the bend trace. The bend trace shows large-amplitude bends (alternating between approximately +50 to −50 degrees) as well as many smaller oscillations. The maximum bend is the difference between the average of the most positive and negative values of the dominant bends in a bend trace.

Recorded images are processed through several steps to produce a spline. Pixels in the original video frames are in greyscale with brightness values between 0 (black) and 255 (white). Each video frame is converted into a binary image using a user-selected brightness threshold, such as 100. All pixels above the threshold are converted to pure white whereas all pixels below it to pure black. The software would automatically vary the selected threshold from −20 to +20% for up to 10 attempts if error-checking algorithms flag the video frame as unanalyzable. In the binary image, all objects smaller than 3000 pixels in area (1% of a 640×480 pixel image) are removed to prevent complications by debris and other artifacts, and all gaps produced by light spots within the worm body are filled in. An edge detection algorithm is used to find the outline of the worm. Subsequently, the raw edge data are smoothed and converted into a series of x-*y* coordinates. These procedures transform a gray image into a binary image in which the worm appears in solid black color (Figure 1A).

#### Head identification

The tail of the worm forms a sharper angle than does the head in the binary image. A custom corner-detection algorithm was developed to search for the sharpest and second-sharpest corners to designate them as the tail and head candidates, respectively. This approach is similar to that used in a previous study [4]. Error-checking mechanisms are built into this process to ensure that corners formed by body bends are not mistaken as either the head or the tail. Head identification in each subsequent frame also uses the head position in its preceding frame as a reference to facilitate the process.

#### Spline generation and centroid determination

The spline is created along the midline of the worm profile by performing a cubic interpolation of midpoints. It is then divided into 12 segments by placing 13 markers at equal intervals, resulting in 11 consecutive angles (Figure 1A), which may be quantified individually to determine bending properties. The centroid position is defined as the averaged position of the 13 markers.

#### Bending properties

*Track-A-Worm* plots bending activities of a worm over the recording period as a *Bend Trace* (**Figure 1C**). The appearance of a bend trace gives the user some general idea about bending properties. The most important use of the bend trace is to serve as the basis for detailed quantifications of bending properties, including *Dominant Bend Frequency*, *Maximum Bend*, and *RMS* (root mean square) bend activity. These quantifications may be based on any of the 11 bends. In addition, *Track-A-Worm* also reports the *Sum of All Bends*.

*Dominant bend frequency* – A worm typically bends with one large dominant motion intervened by small oscillations. A Fourier transform of a bend trace is performed to obtain the frequency spectrum, in which the most prominent frequency peak is defined as the *Dominant Bend Frequency*. For example, a plot of the frequency spectrum for the bend trace shown in **Figure 1C** gives the frequency spectrum with a dominant bend frequency of approximately 0.4 Hz (**Figure 1D**).

*Maximum Bend* – It is often intuitive and useful to quantify the amplitude of the dominant bends in a bend trace. The Maximum Bend is the difference between the averages of the negative and positive values of the dominant bends in a bend trace (**Figure 1E**). This measure is only useful in worms that exhibit organized bending behavior. If the bending behavior is very chaotic without regular large-amplitude bends, this parameter is difficult to quantify.

*RMS bending activity* – Although the Maximum Bend described above is an intuitive measure of bending angles, it is somewhat subjective and, most importantly, does not measure all bending activity. Besides the maximum bends, a bend trace is usually decorated by numerous small oscillations (**Figure 1C**), which are not included in the analysis of the maximum bends. RMS bend serves as an objective, sensitive, and accurate measure for the overall bending activity according to the formula:
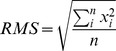
where *n* is the total number of points and *i* refers to each individual point. A limitation with the RMS parameter is that it does not distinguish between low-amplitude high-frequency versus high amplitude low-frequency behavior.

*Sum of All Bends* – The Sum of All Bends is a useful metric for quantifying the bending behavior of an entire worm. It is simply the sum of all the 11 bends of a worm averaged over time. Note that unlike the standard bend trace, the Sum of All Bends metric uses the absolute values of the bends, so the direction of the bend is not considered.

#### Movement path

**Figure 2:**
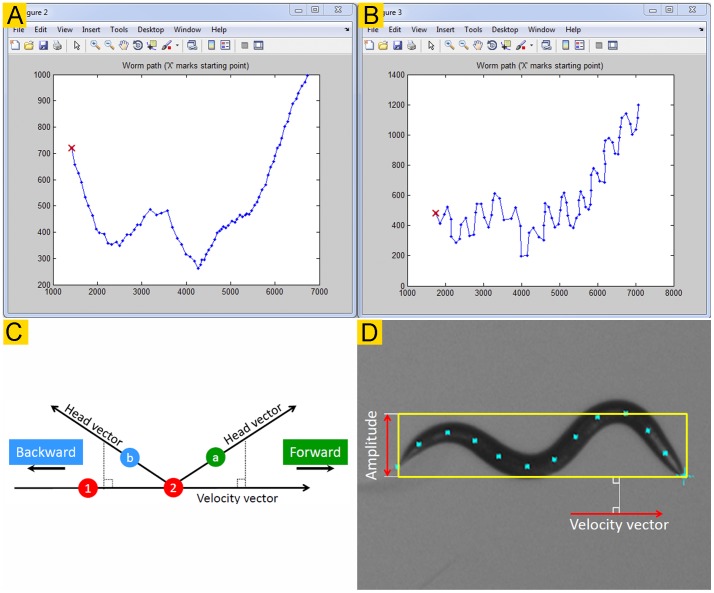
Reconstruction of travel path, and determination of movement direction and body amplitude. **A & B**. The traveling paths of a wild-type worm reconstructed based on the positions of the centroid (**A**) and the nose/1^st^ marker point (**B**). **C**. Directionality is determined by comparing the directions of a velocity vector and a head vector. The velocity vector is obtained by connecting the centroid positions of two consecutive frames (red circles “1” and “2”) whereas the head vector by connecting the current centroid and head positions. The green circle labeled “a” and the blue circle labeled “b” show that the head vector is projected to the positive and negative sides of the velocity vector, respectively. The worm is moving forward if the projection of the head vector onto the velocity vector is positive. **D**. The amplitude is determined by first finding the velocity vector, and then drawing a box in alignment with the velocity vector to enclose the widest points of the spline with the width of the box being the worm amplitude.

It is frequently useful to trace the locomotion path of a worm in the analysis of mutant behavior. *Track-A-Worm* can reconstruct the travel path of a worm according to the positions of either the centroid or any of the 13 markers along the spline by combining stage movement data with consecutive worm images (**Figure 2A, B**).

#### Speed, distance, and direction

These metrics are typically based on the positions of the centroid over successive images although any of the 13 markers along the spline may be used. The *Speed* and *Distance* functions measure the average speed (µm/sec), the total distance travelled (both forward and backward) as well as the net distance traveled (straight-line distance between the first and last positions of the worm) over the recording period. The total distance travelled may be determined according to the positions of either the centroid or any of the 13 markers along the spline whereas the net distance traveled may be determined only according to the centroid positions. Directionality is determined by comparing a *velocity vector* formed by connecting the last and current centroids in two consecutive frames and a *head vector* formed by connecting the current centroid and nose [1]. If the projection of the *head vector* onto the *velocity vector* is positive, the worm is determined to be moving forward and vice versa (Figure 2C).

#### Amplitude

Amplitude is another measure of worm curvature. It is determined by first finding the velocity vector as described above and then measuring the width of a rectangular box drawn in alignment with the velocity vector and just large enough to contain all spline points (Figure 2D). The software also calculates the ratio of the amplitude over worm length (*A/L*) to account for the effects of worm size variation on the measurement.

## Results

**Figure 3:**
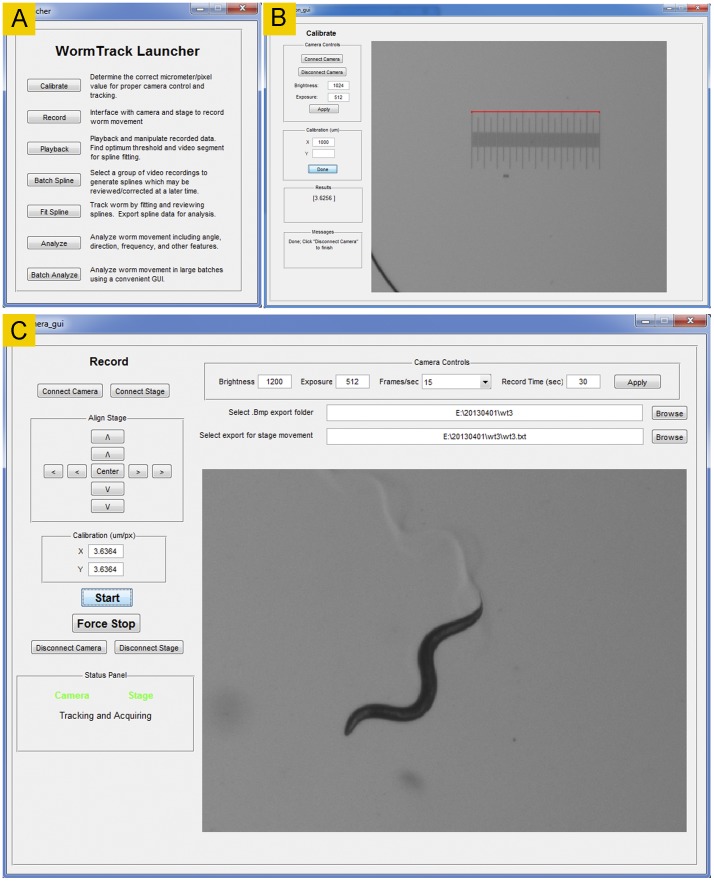
The *Launcher, Calibrate*, and *Record* modules. **A**. The *Launcher* interface is for launching the various modules of *Track-A-Worm*. **B**. The *Calibrate* module determines the correct conversion factor from pixels to micrometers. **C**. The *Record* module is used to track and record a freely moving worm.

*Track-A-Worm* includes seven different modules, including *Calibrate, Record, Playback, Batch Spline, Fit Spline, Analyze*, and *Batch Analyze*. Each module has its own graphic user interface and may be launched from the software launcher window (**Figure 3A**). The section below introduces the graphic interfaces and functions of the various modules. A detailed user guide may be found in **File S2**.

### Calibration

Prior to recording images, the correct conversion factor from pixels to micrometers should be determined. This is done with the Calibrate module (**Figure 3B**).

### Record

The Record module (**Figure 3C**) receives worm position information from the camera, sends commands to re-center the stage at 1-sec intervals, and outputs sequential images to a user-designated folder. It also saves a text file containing stage movement information. The user may define the conditions for recording the images, including brightness, exposure level, frame rate (for Sony XCD-V60, choices are 1, 3, or 15/sec), and recording duration. Before the recording, it is important to specify the file folder and the file name for the recording, center the stage, and enter the correct *XY* calibration values.

### Playback

**Figure 4:**
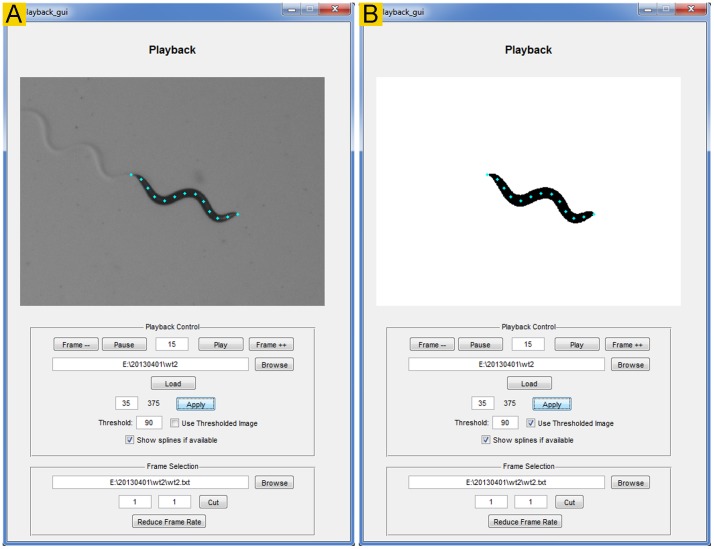
The *Playback* module. This module is for viewing recorded images (either as a movie or as separate frames), cutting out undesired frames, and evaluating the brightness threshold for binary conversion. It may display either original (**A**) or binary (**B**) images. It also has the option to display the 13 markers after spline fitting.

The *Playback* module (**Figure 4**) allows the user to view recorded sequential images (original or binary) as a movie, cut out undesired frames, and assess the brightness threshold for binary conversion. The cutting function may be used to extract sequential video frames from any time period but is typically used to eliminate the first few seconds of a recording because some cameras automatically adjust brightness and exposure during that period to achieve optimal recording conditions. After spline fitting, splines may also be overlaid on the images.

### Fit Spline and Batch Spline

**Figure 5:**
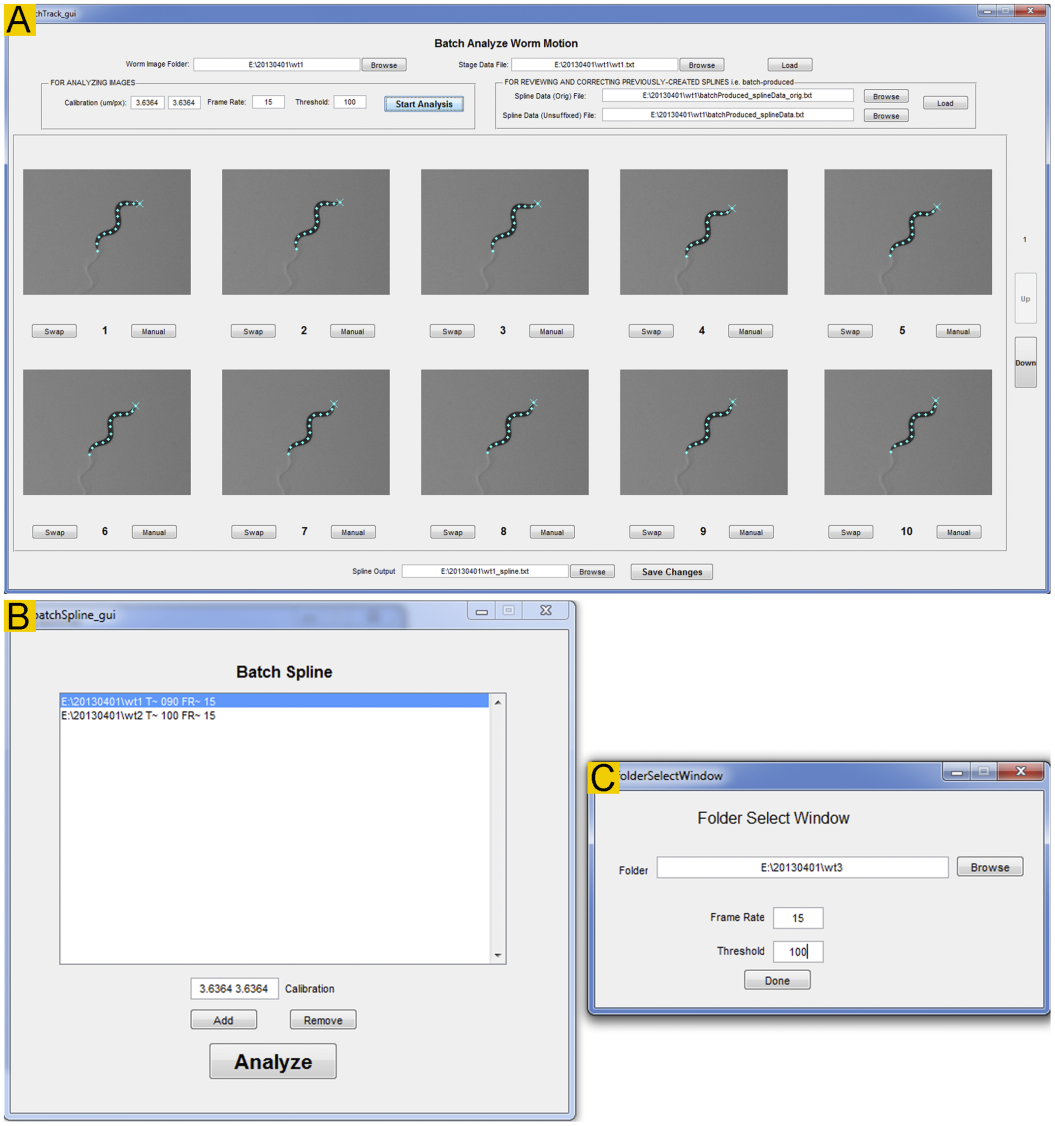
The *Fit Spline* and *Batch Spline* modules. **A**. The *Fit Spline* module is used for automatic spline fitting, and for user verification and correction of the spline fitting and head identification results. The blue “×” sign indicates the head position while the 13 blue dots mark the deduced spline (not shown). If the tail was mistaken as head during the automatic analysis, the user may swap them simply by clicking the “Swap” tab. This action swaps the head and tail for the current as well as all the subsequent frames. This rule is applied because head identification in every subsequent frame uses the preceding frame as a reference during the automatic analysis. If the spline was generated incorrectly, the user may click the “Manual” tab to open the frame in a new window to correct it. **B & C**. *The Batch Spline module* is used for automatic spline fitting and head identification for multiple files in a batch. A small record selection window (**C**) pops out when the *Add* button in **B** is clicked.

The *Fit Spline* module (**Figure 5A**) has two main functions: (1) automatic spline fitting and head identification, and (2) user-involved verification and correction of the automatically generated results.

In the first step, the software automatically determines the worm outline, identifies the head, deduces the spline from the worm outline, and places 13 markers along the spline. To use this function, it is important to enter a reasonably optimal brightness threshold for binarization. The appropriate threshold value may be assessed using the *Playback* module. In most cases, *Track-A-Worm* may extract the desired information on a single attempt. Occasionally, however, the use of a non-optimal threshold with changing lighting conditions or an unusual worm body-shape (e. g. an Ω shape) could prevent this from happening. In such cases, *Track-A-Worm* automatically adjusts the brightness threshold by up to 20% in either direction, and repeats the analysis until either an acceptable spline has been extracted or a total of 10 attempts have been made. Images that cannot be successfully analyzed after 10 attempts are left out for semi-automatic analysis in the next step. Upon the completion of analyzing all the frames of a recording, 13 markers and a head label (indicated by a “x” sign) are displayed over each worm image (**Figure 5A**). The results of this step are saved as two spline files, including one with stage movement and calibration (pixel-to-micrometer conversion) compensations and one without such compensations.

In the second step, the user is prompted to verify the results of the first step and to correct any mistakes. This step is necessary because errors could occur and information extraction could fail during the previous step. To facilitate the process, *Track-A-Worm* displays 10 frames in two separate rows at a time, and allows the user to advance 5 frames (1 row) on a single mouse click (**Figure 5A**). With the recommended 1920×1080 resolution display, it is easy to tell whether the head has been correctly identified, and whether a spline with 13 markers (indicated by blue dots) has been deduced. Any error may be easily corrected by clicking the “Swap” or “Manual” button below the image.

The first step, automated spline fitting, is computation-intensive but requires little user intervention. The *Batch Spline* module (**Figure 5B, C**) allows the user to choose multiple recordings for the automatic analyses without personal attention. The generated results are then subjected to the same above-described user-dependent verification and correction. For example, the user can analyze many recordings overnight, and proofread them the following day.

### Analyze and Batch Analyze

*Track-A-Worm* has two graphic interfaces for analyses: *Analyze* and *Batch Analyze*. In both cases, the spline files and corresponding stage files are used as input to generate quantitative data. Corresponding stage files are chosen automatically when specific spline files are selected.

**Figure 6:**
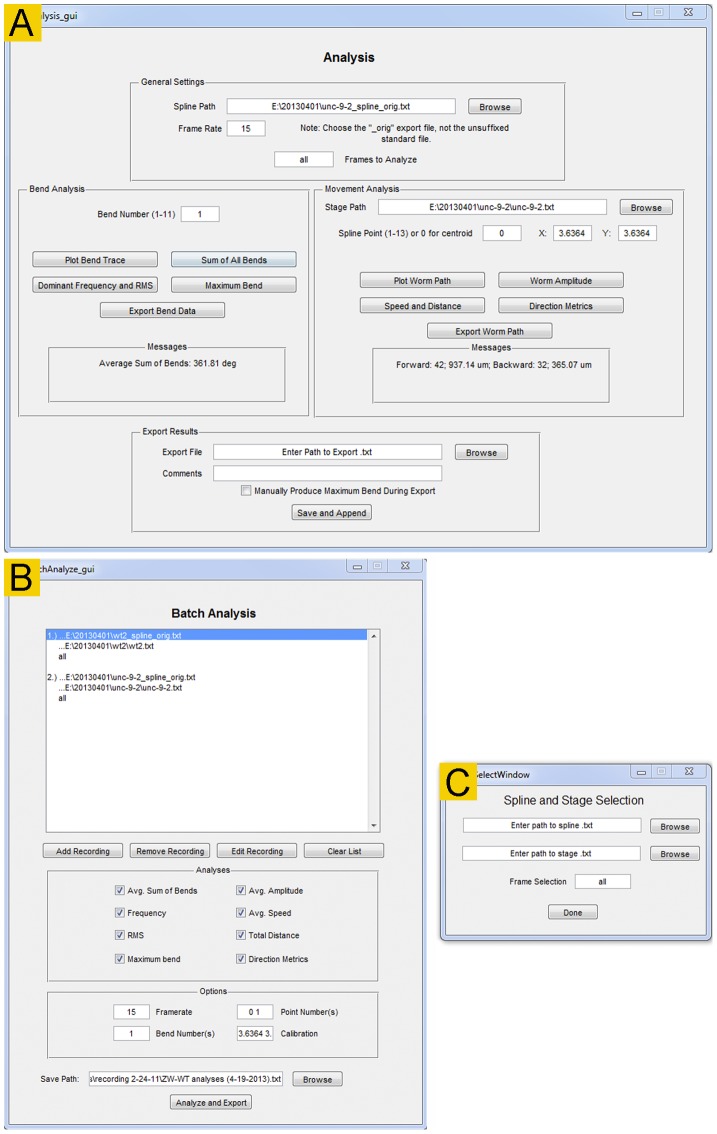
The *Analyze* and *Batch Analyze* modules. These two modules are used to quantify worm movement and bending behavior. **A**. The *Analyze* module is for quantifying bending and movement behavior of one worm at a time although the results may be appended and saved in a single file. **B**. In the *Batch Analyze* module, multiple recordings can be quantified and exported to one file. This is ideal for rapidly comparing and summarizing large groups of worms. All functionality of the *Analyze* module except for graph plotting is replicated in the *Batch Analyze* module.

*Analyze* (**Figure 6A**) is for overviewing and analyzing recordings individually. The quantitative parameters are divided into two categories: *Bend Analysis* and *Movement Analysis*. The functions of *Bend Analysis* include plotting the bend trace and bend frequency spectrum, quantifying the magnitude and frequency of maximum bends, quantifying the sum of all bends averaged over time, and reporting bending activities as RMS and maximum bend for any selected bend (1 to 11). The functions of *Movement Analysis* include reconstructing the worm travel path, reporting the worm amplitude, calculating the speed and distance of movements, and calculating the duration and distance of forward and backward movements. All of these parameters except for worm amplitude and directionality may be determined according to the positions of either the centroid or any of the 13 markers. Bend analyses are performed at the full frame rate of the recording (typically 15 frames per second) whereas movement analyses are performed at either 1 or 3 frames (depending on the stage) per second to accommodate the time required for stage movements. The lower frame rate still appears to be sufficient for accurate quantification of the movement metrics. The results of analyses are displayed in the *Messages* boxes, and may be also exported and continually appended to existing files.

*Batch Analyze* (Figure 6B) is for analyzing data from multiple experiments in a batch. It can perform all the functions described for the Analyze module except for the plotting of bend trace, bend frequency spectrum, and worm travel path.

## Discussion

When deciding what parameters were to be quantified or characterized by *Track-A-Worm*, we referred to parameters found in several leading worm trackers [1], [2], [5] as well information available about their current versions at the developers' websites (http://wormlab.caltech.edu/publications/download.html; http://www.mrc-lmb.cam.ac.uk/wormtracker/), and considered the potential common needs of *C. elegans* research labs. The current version of *Track-A-Worm* allows the quantification of most of the parameters found in the other systems. In addition, we added RMS as a measure for worm bending activities. As described earlier, worm bending activities typically include large-amplitude low-frequency sweeps as well as many apparently random small oscillations. Previous worm trackers focused on only the large-amplitude bends. We thought that behavioral abnormalities of some mutants might be reflected by a change in the smaller oscillations and decided to use RMS as a measure for all bending activities. Indeed, we found that several mutants differed significantly from wild type in head bending when measured by either RMS or the maximal bends [23]–[25]. The quantification for both maximum and RMS bends is meaningful because some mutants might exhibit changes in just one of the two parameters.

The deduced spline forms the basis for quantifying worm bending properties. However, it is difficult for an automated worm tracker to completely avoid errors because some unusual body shapes (e. g. an Ω or O shape) or poor lighting conditions could present a challenge to correctly deduce the spline. Therefore, it is important to include a feature allowing the users to verify the deduced spline and to correct any errors. A strong feature of *Track-A-Worm* is that it contains a graphic interface allowing the user to review 10 frames at a time and to correct with ease any errors introduced during the automatic analyses. This feature ensures that subsequent analyses are based on high-quality raw data.

*Track-A-Worm* could potentially be improved by adding some new features. For example, it could potentially be combined with optogenetic stimulation and calcium imaging of selected neurons, which are features found in several other worm trackers [4], [15], [17], [21], [26]. Algorithms for resolving challenging body shapes (*e. g.* an Ω shape) or for tracking worms moving in 3-D environment (*e. g.* swimming), as have been done by others [27], [28], could potentially be added. Additional hardware may be supported, further broadening the versatility of *Track-A-Worm*. The fact that the code of *Track-A-Worm* is open source makes it convenient to add new features to serve various needs.

## Supporting Information

File S1 **Comparisons of locomotory and bending behavior between wild type and** ***unc-9(fc16)*** **mutant.**
(PDF)

File S2 ***Track-A-Worm*** **user manual.**
(PDF)
