# Supplementary material for: Track-A-Worm, An Open-Source System for Quantitative Assessment of C. elegans Locomotory and Bending Behavior
Source: PLoS One. 2013 Jul 26;8(7):e69653. doi: 10.1371/journal.pone.0069653 (PMC3724933; doi:10.1371/journal.pone.0069653)
Supplement: File S1 — Comparisons of locomotory and bending behavior between wild type and unc-9(fc16) mutant. (PDF) [file pone.0069653.s001.pdf]

Quantitative comparisons of locomotory and bending behavior between wild-type and *unc-9* mutant worms

To further demonstrate the usefulness of *Track-A-Worm*, bending and movement analyses were performed for the wild type and *unc-9(fc16)*, which is a putative null. While the software may perform bend analyses for any of the 11 bends and movement analyses for the centroid or any of the 13 markers, only the first bend was selected for bend analyses, and only the centroid and first marker were selected for movement analyses. The table below shows the 'means ± SE' values for the selected parameters. Some of the table contents are plotted in **Figure S1** as bar graphs.

Table S1. Comparisons of locomotory and bending behavior between wild type and *unc-9(fc16)* mutant

Parameters	WT	*unc-9*	*p* value
Average sum of all bends (degrees)	309.6 ± 4.8	350.2 ± 4.7	< 0.01
Dorminant bending frequency (Hz)	0.45 ± 0.01	0.35 ± 0.05	NS
RMS bends (degrees)	29.4 ± 0.5	38.2 ± 0.6	< 0.01
Maximum bends	91.9 ± 1.9	97.5 ± 1.8	< 0.05
Worm amplitude	239.3 ± 6.9	403.2 ± 7.6	< 0.01
Worm amplitude/length	0.25 ± 0.00	0.45 ± 0.01	< 0.01
Centroid speed (µm/sec)	174.6 ± 7.1	42.2 ± 2.3	< 0.01
Marker #1 speed (µm/sec)	279.0 ± 7.1	224.5 ± 5.1	< 0.01
Total distance (centroid)	4306.8 ± 175	977 ± 73.5	< 0.01
Total distance (marker #1)	6882.3 ± 174.7	5164.1 ± 245.4	< 0.01
Number of steps (frames) forward	71.1 ± 0.9	43.3 ± 2.2	< 0.01
Number of steps (frames) backward	2.9 ± 0.9	25.7 ± 2.2	< 0.01
Distance forward (µm)	4181.9 ± 201.9	640.2 ± 56.0	< 0.01
Distance backward (µm)	124.9 ± 41.8	337.0 ± 39.2	< 0.01
% of time on forward movement	0.97 ± 0.01	0.66 ± 0.03	< 0.01
% of time on backward movement	0.03 ± 0.01	0.34 ± 0.03	< 0.01
Forward duraton (sec)	23.7 ± 0.3	14.4 ± 0.7	< 0.01
Backward duration (sec)	0.98 ± 0.31	8.6 ± 0.7	< 0.01
Forward speed (µm/sec)	175.8 ± 7.0	44.1 ± 3.0	< 0.01
Backward speed (µm/sec)	131.5 ± 17.9	38.0 ± 2.3	< 0.01

n = 15 in both groups. Un-paired *t*-test was used for statistical comparisons.

Show are bending perperties of bend #1, overall speed and total distance of the centroid and marker #1, and directional movements based on the centroid.

The Maximum Bend was determined using a threshold of +10 and +10 degrees.

The data in the last six lines were calculated as follows:

% of time on forward (or backward) movement =
Number of forward(or backward) frames/Total number of frames

Forward (or backward) duration = Number of forward (or backward) frames/3

Forward (or backward) speed = Distance forward (or backward)/Forward (or backward) duration

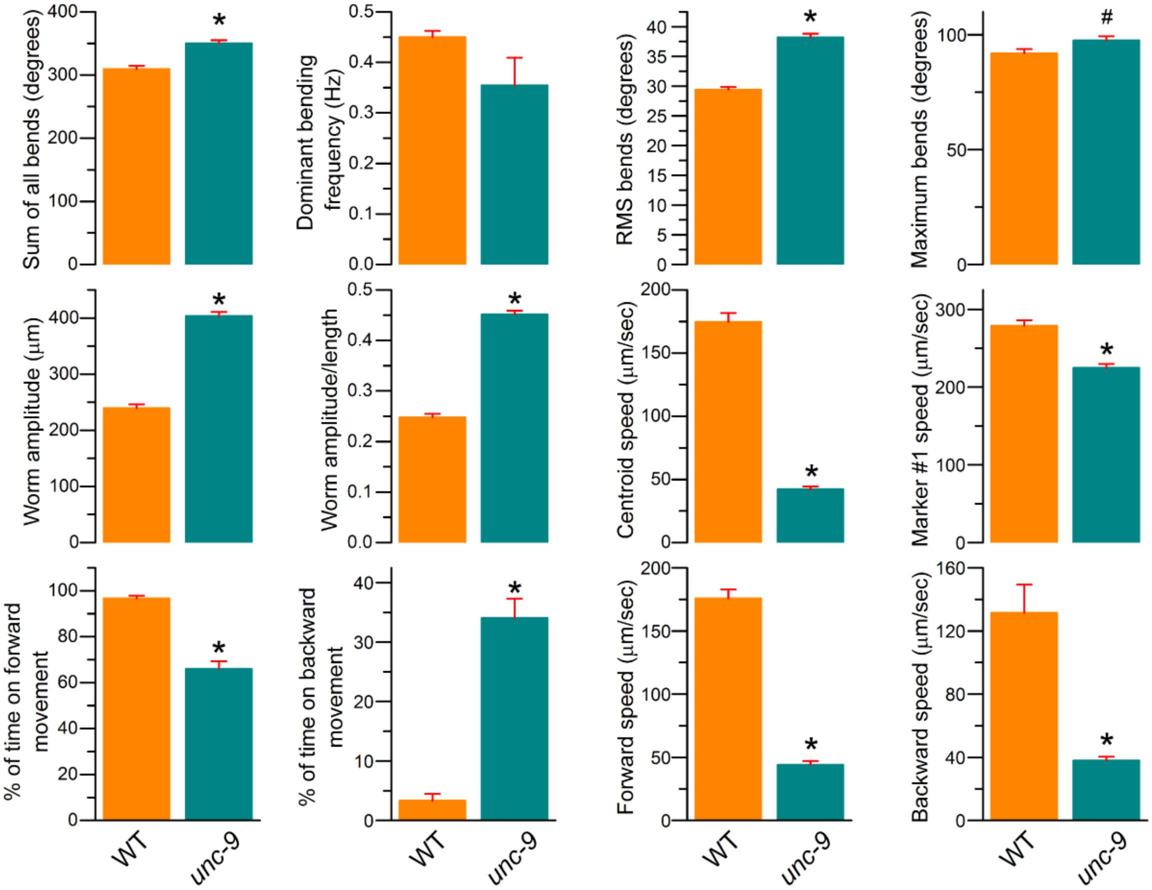

Figure S1. Comparisons of locomotory and bending behavior between wild-type and *unc-9(fc16)* mutant. The pound sign (#) and the asterisk (*) indicate '$p < 0.05$' and '$p < 0.01$', respectively, compared with the wild type (WT).

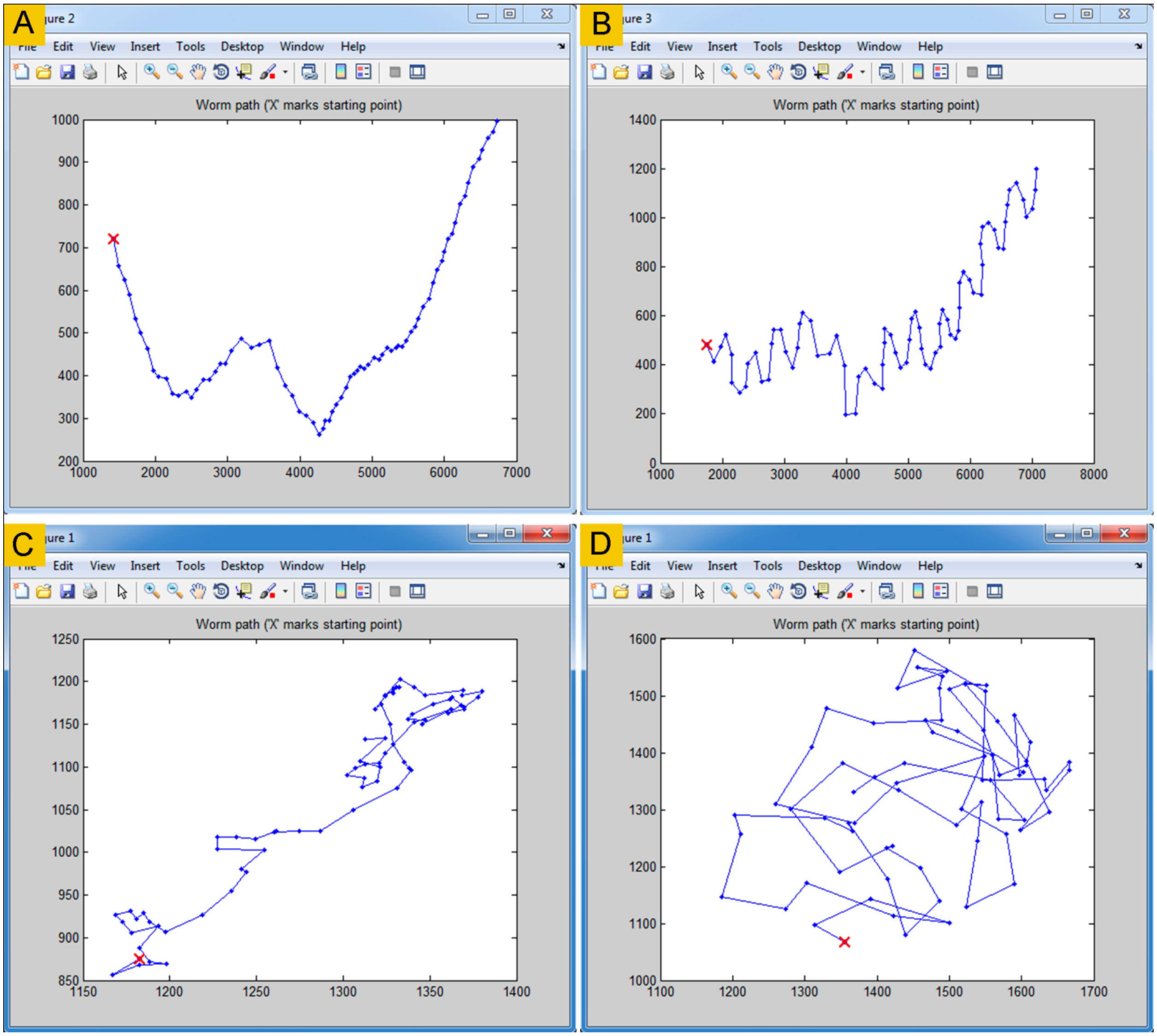

Figure S2. Worm paths of a wild-type worm and an *unc-9(fc16)* mutant worm. A & C. Worm paths based on the positions of the centroid of wild type (**A**) and *unc-9* mutant (**C**). **B & D.** Worm paths based on the positions of the first marker of wild type (**B**) and *unc-9* mutant (**D**). The red 'x' marks the starting point.
