# Supplementary material for: Track-A-Worm, An Open-Source System for Quantitative Assessment of C. elegans Locomotory and Bending Behavior
Source: PLoS One. 2013 Jul 26;8(7):e69653. doi: 10.1371/journal.pone.0069653 (PMC3724933; doi:10.1371/journal.pone.0069653)
Supplement: File S2 — Track-A-Worm user manual. (PDF) [file pone.0069653.s002.pdf]

Track-A-Worm

User Manual

System Setup

Track-A-Worm requires the following major hardware components:

- A stereomicroscope
- A digital camera
- An *x-y* motorized stage
- A PC computer installed with Windows 7 (32-bit) and Matlab (including tool boxes for image processing, image acquisition, instrument control, and signal processing).

The hardware used in our system is shown in **Figure S1**. For alternative hardware, please consider choosing a fast PC with a large monitor (*e. g.* 24-inch), an *x-y* stage with travel distances longer than the diameter of the petri dish used in worm tracking, and a camera that has driver support for Matlab. Compatibility with 64-bit versions of Matlab and Windows operating systems has not been tested. Matlab drivers for the camera and stage are designed to be modular. Therefore, only a few functions must be replaced in *Track-A-Worm* software to accommodate different hardware.

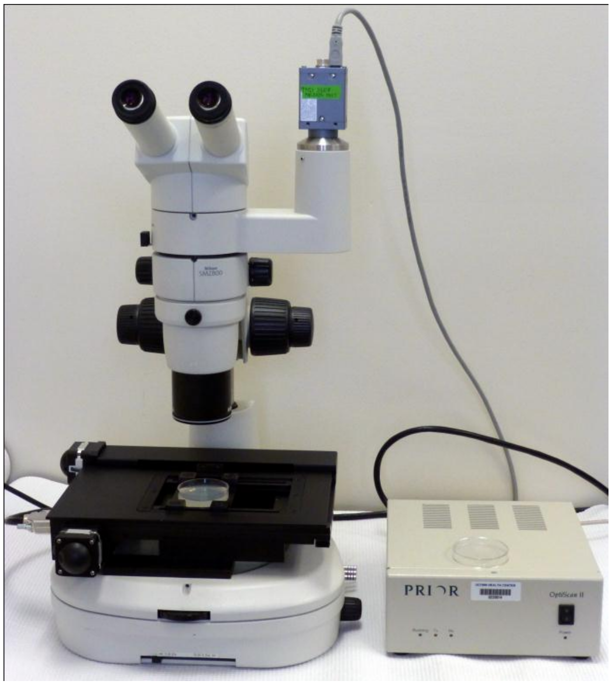

Figure S1. *Track-A-Worm* hardware setup. The major components include (1) a zoom stereo microscope (SMZ-800, Nikon), (2) a black-and-white CCD camera (XCD-V60, Sony), (3) a *x-y* motorized stage (Optiscan™ ES111, Prior Scientific, Rockland, MA, USA) with a stage controller (Optiscan II), a universal specimen holder (H473) and a stage stand (H413), and (4) a PC computer (not shown) installed with Windows 7 (32-bit) and Matlab R2012b. The stage has a travel range of 126 mm X 76 mm with a minimum step size of 1 µm.

Software Installation

Copy *Track-A-Worm* program files to a directory of your choice. Create a shortcut to "Matlab.exe" (typically in C:\Program Files\Matlab\bin\Matlab.exe) on your desktop. Right-click the newly created icon and modify the field "Start In" to reflect the directory where *Track-A-Worm* is located. *Track-A-Worm* can now be launched by double-clicking the shortcut icon. We suggest that you rename this icon to *Track-A-Worm*.

Operating Instructions

File structure

Track-A-Worm saves files to and looks for them in a predetermined hierarchy. Before the recording, you need to manually create a main folder for saving your data, such as '20130401' based on the date of the recordings. Suppose that you would like to use 'wt1' as the base name for the first recording of wild-type worms, you simply need to type '\wt1' after the folder name (*e. g.* \20130401\wt1) into a specific open box in the *Record* module before the recording (details in a later section). A subfolder named 'wt1' would be created automatically when the recording is started. This subfolder contains (1) recorded image files that were automatically named as 'img001.bmp, img002.bmp, img003.bmp, etc'; (2) a subfolder named 'removed' containing removed files. This folder is automatically generated when the *Cut* function in *Playback* module is used to remove some files; (3) an active stage file named as 'wt1.txt', and another stage file named as 'wt1_orig.txt', which is the original stage file renamed after performing the *Cut* function in the *Playback* module. The stage files contain stage movement information; and (4) two spline files generated using the *Batch Spline* module, including 'batchProduced_splineData.txt' and 'batchProduced_splineData_orig.txt'. Upon finishing spline inspection and correction using the *Fit Spline* module, two spline data files are automatically generated and saved in the '20130401' folder as 'wt1_spline.txt' and 'wt1_spline_orig.txt', which contain the *x* and *y* coordinates (in micrometers) of the spline over time with and without stage movement and calibration compensations, respectively. In other words, the unsuffixed file is both stage-compensated and calibrated, while the "orig" file is in raw pixels only. These two files are saved in this folder so that the user may find spline files of all the recordings of related experiments in the same place. The two splines generated using the *Batch Spline* module are saved in the 'wt1' subfolder instead of the '20130401' folder because the spline fitting and head assignment need to be verified by the user at first.

To maintain the integrity of the folder and file structures, the user should avoid changing either the folder hierarchy or folder/file names using Windows Explorer. When it is necessary to do so, be sure to make corresponding changes to all the related folders and files to reflect the convention described above. A graphical user interface typically contains several fields displaying the path and name for all the related files. However, only the path and name of the base file have to be entered manually. Upon pressing "Enter", the path and name of the related files are automatically entered or updated with the updated fields flashing green momentarily. This feature is included to save time and prevent user errors.

To run *Track-A-Worm*, click the desktop icon that you created earlier. The first window that appears on the screen (**Figure S2**) allows the user to launch various modules from it, including *Calibrate, Record, Playback, Batch Spline, Fit spline, Analyze*, and *Batch Analyze*. The procedures for using each module are described below.

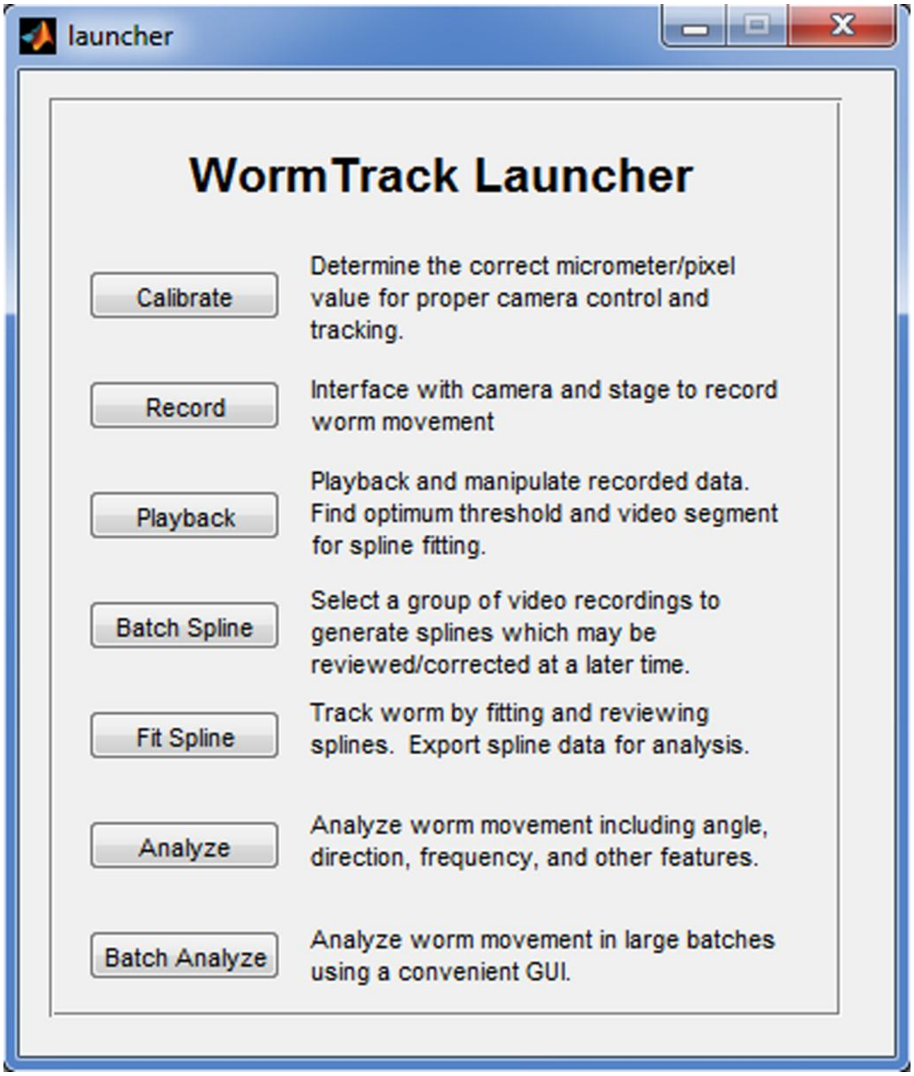

Figure S2. *Track-A-Worm* launcher.

Calibration

Prior to making a recording, the correct conversion factor from pixels to micrometers needs to be determined using the *Calibrate* module (**Figure S3**). The *Calibrate* module receives input from the camera. Operation is as follows:

1. Place the stage under the microscope objective, and align its *x* axis straight relative to the camera. It is important to maintain the stage in the same orientation during subsequent image recordings although the exact location of the stage does not matter.
2. Click *Connect Camera* and wait for the camera to start up.
3. Place a stage micrometer under the microscope, and align it in either the *x* or *y* direction.
4. Adjust microscope magnification to a desired level.
5. Click first on one end of the micrometer and then on the other end to observe a straight red line drawn between them.
6. Enter the actual distance spanned by the drawn line into the *x* or *y* box under *Calibration (µm)*. You may leave the other box blank (*e. g.* leaving the *y* box blank when calibrating for *x*);
7. Click *Done* to observe the calibration displayed in the *Results* box.
8. Repeat the procedures to perform calibration for the other direction, which is necessary.

Click *Disconnect Camera* before exiting the module. Write down the calibration results so that you may enter them into the other modules at a later time. You do not need to recalibrate if you are going to use the same microscope magnification settings in subsequent experiments.

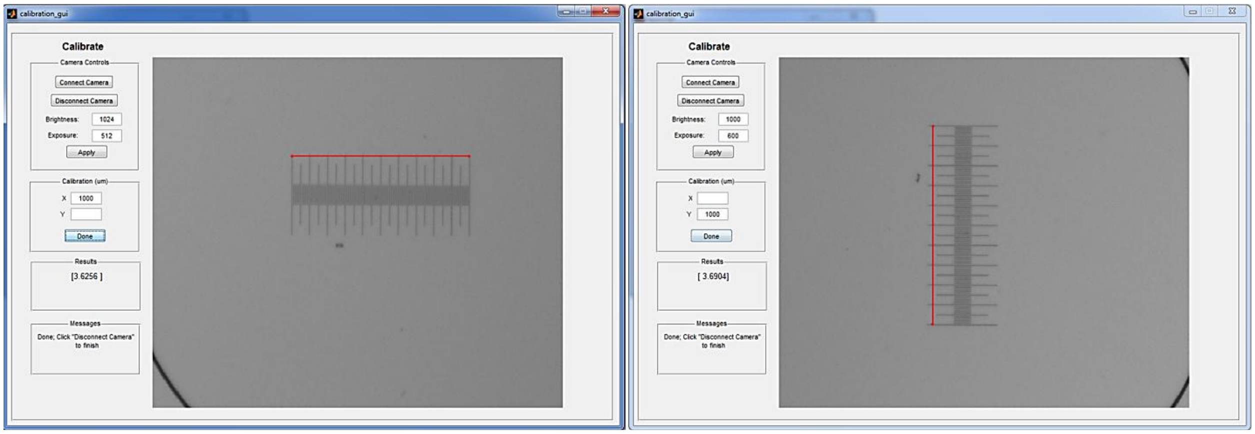

Figure S3. Calibrate module. This module is used to determine the correct conversion factor from pixels to micrometers for the *x*-axis and *y*-axis.

Record

The *Record* module (**Figure S4**) receives worm position information from the camera, sends commands to re-center the stage at 1-sec intervals, and outputs sequential images to a user-designated folder in BMP format. It also saves a text file containing all the stage movement information.

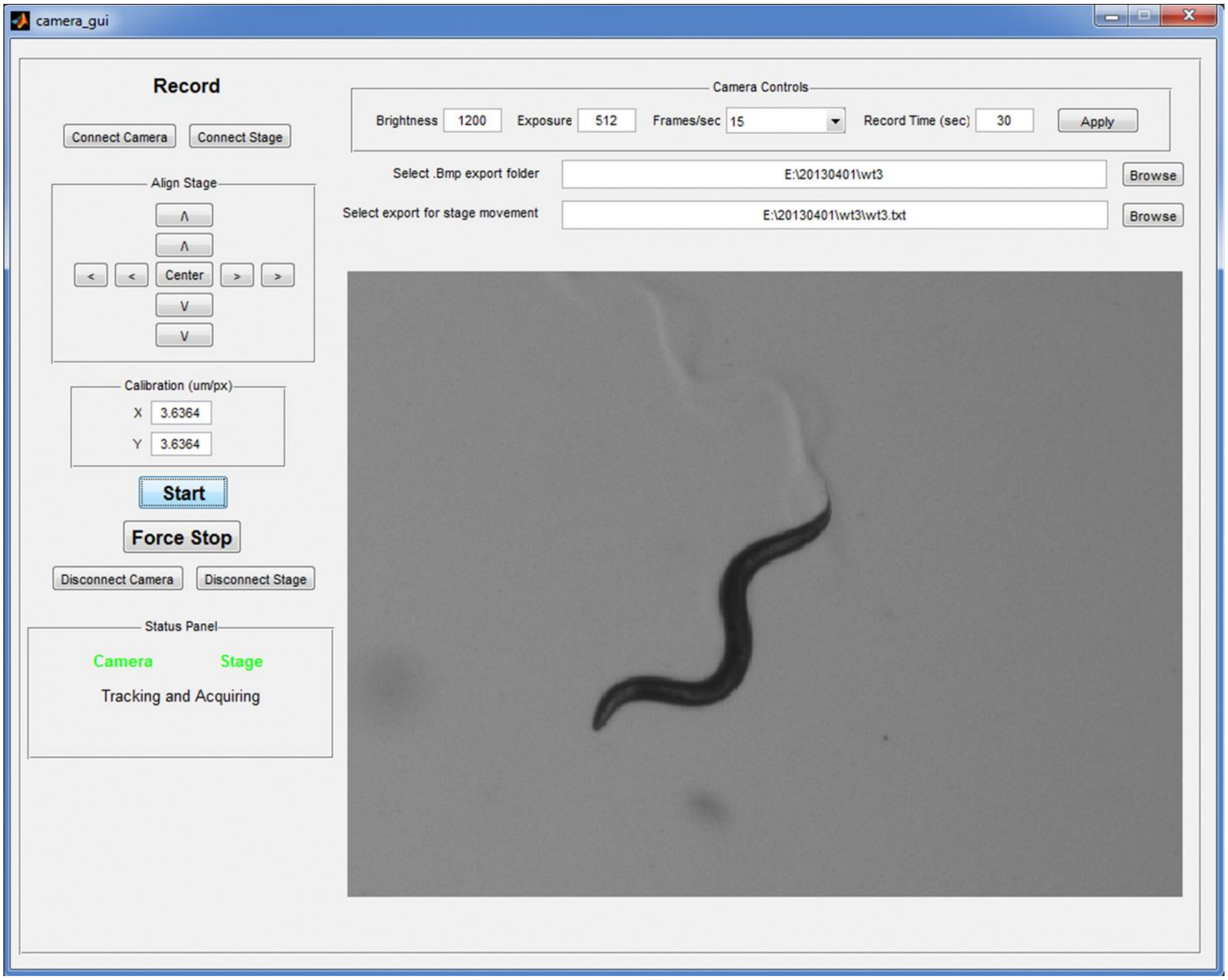

Figure S4. Record module. This module is used to record worm images. To add a new recording (*e. g.* 'wt3') to the same folder (*e. g.* '20130401') after finishing one recording (*e. g.* 'wt2'), the user only need to change '2' to '3' in the open box before the first *Browse* button and press the *Enter* key to observe that the stage movement file name is updated automatically. A subfolder named as 'wt3' is automatically generated at the start of the recording.

A typical workflow is as follows:

1. Click *Connect Camera* and wait a few seconds for the *Camera* indicator in the top-right corner to change from red to green.
2. Click *Connect Stage* and wait a few seconds for the *Stage* indicator in the top-right corner to change from red to green.
3. Click *Center* to ensure that the stage is at its central position.
4. Enter the *x* and *y* calibration values found in the *Calibrate* module.
5. Adjust the relative brightness level, exposure, frame rate, and recording time settings as needed. You must click *Apply* to make the new settings to take effect. The brightness should be set to such

a level that the worm appears as a dark grey color with good solidity as shown in **Figure S4**. The frame rates that may be chosen are 1, 3, 5 and 15. When choosing a frame rate, the user should consider about potential stage limitations. Some stages may be unable to initiate and complete a movement at a sufficiently fast speed, and thus resulting in image blurring due to stage movements. There is no such problem with the Prior stage shown in **Figure S1**.

6. Select the BMP export folder and enter the base file name for the recording. The corresponding folder names in the *Select export for stage movement* are entered automatically.
7. Manually (by hand, not using the *Align Stage* buttons) move the stage to find the worm. Remember to maintain stage orientation as you move it. When the worm is within view, click the *Start* button. You may observe automatic adjustment of the exposure conditions for the first couple of frames. The Sony XCD-V60 camera takes a few seconds to begin recording after the *Start* button has been clicked. During this delay period, the worm may move away from the center of the imaging field. You may manually move the stage within a couple of seconds immediately after clicking the *Start* button but before the tracking has actually started to ensure the worm is still in the camera field. However, as long as the worm has a small part in the imaging field at the moment when the camera becomes ready, it can be identified reliably and moved to the center of the field automatically. A faster-starting camera would improve this aspect of the user experience.

After the designated recording period is over, the *Record* module will save the files. The *Status Panel* will indicate when the process is completed. To begin a new recording, you must click *Connect Camera* and update the base file name (e. g. change ‘wt1’ to ‘wt2’) and, if necessary, change the folder name.

Canceling a recording -- It is sometimes necessary to terminate a recording before it ends. To do so, click on the MATLAB command window to make it active, and then press CTRL-C. A series of error messages will appear, and the recording will be canceled. You will then need to click *Disconnect Camera* and *Connect Camera*. This returns you to Step 3.

Playback

The *Playback* module (**Figure S5**) allows the user to load a recording for playback, evaluate the threshold for binary conversion, specify a frame or frames to display, and cut out undesired frames. The user interface of this module includes three sections: *Video viewer, Playback Control*, and *Frame Selection*. The video viewer can display images in either original or binary format. To use this module, the user first needs to load a recording using the *Browse* button under *Playback Control*.

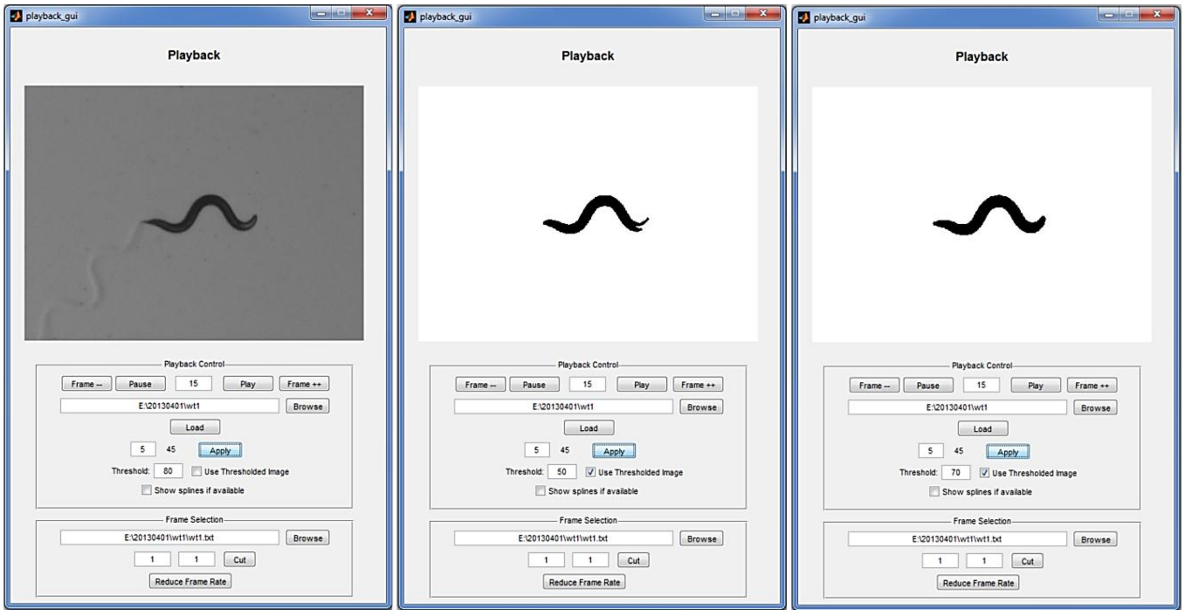

Figure S5. Playback module. *Left*: An original worm image is displayed. *Middle:* The threshold was set too low, resulting in an incomplete worm silhouette in the binary image. *Right*: The entire worm appeared as solid black after the threshold was increased.

The open box between the *Play* and *Pause* buttons is for choosing a relative playback frame rate. The actual playback frame rate also depends on the speed of the computer. The *Frame++* and *Frame−−* buttons are used to move forward and backward a single frame. The current frame number (the open box) and the total number of frames are displayed below the *Load* button. The frame for display can be selected by typing into the open box and clicking *Apply*. The *Threshold* box allows the user to enter a threshold value for binary conversion. Effective threshold values vary depending on lighting conditions. A higher threshold should be chosen for brighter images, and vice versa. If there is a strong contrast between the worm and background, then a wide range of thresholds may work. Conversely, only a narrow range of thresholds may work. If the brightness level is set too low, portions of the worm may blend into the background, resulting in an incomplete silhouette (**Figure S5**, *middle*). If the brightness level is set too high, shadow around the worm may be included as part of the worm, resulting in a wider worm body in the binary image. The worm in the binary image should appear in solid black color and have a shape closely matching that of the original worm (**Figure S5**, *right*). *Track-A-Worm* has built-in mechanisms to automatically adjust contrast, eliminate background noise, and fill in “holes” in the worm body so that a well-represented worm profile can generally be produced. The small check box before the *Use Thresholded Image* determines whether the original or binary images are displayed in the *Video Viewer*. The small check box before *Show spline if available* determines whether the 13 markers representing the spline are overlaid on the worm.

When selecting frames using the Frame Selection section, it is important to remember that **stage movement data are tied to the image sequence.** Therefore, to cut a specific portion of the recording for subsequent analyses, the stage movement file must be updated. When entering the frame numbers for the lower and upper cut edges, **the number for the lower cut edge should be *(fn + 1)* whereas that for the upper cut edge *fn*, where *f* is the frame rate and *n* is an integer.** Therefore, if the frame rate is 15

and you want to keep the data from frame 20 to 80, you should actually select from frame 31 to 75. Otherwise, stage movement timing becomes disrupted. The action of cutting creates a subfolder within the data folder named as ‘removed’. The updated stage file reflecting the selected data is saved using the original file name whereas the original stage file is renamed by appending ‘_orig’ to the file name. **Therefore, subsequent analyses should be carried out on the unsuffixed stage file.** The ‘_orig’ stage file and the contents of the ‘removed’ folder can be used to manually restore the data folder back to its original state. Note that the path and stage file name is automatically entered in the *Frame Selection* section although a *Browse* button is available to override the default choice.

Fit Spline

The Fit Spline module (**Figure S6)** takes the recorded images and the stage file as input to produce two spline files. Both spline files display *x-y* positions of the 13 spline markers in a spreadsheet format. One file (*e. g.* ‘wt-1_spline.txt’) contains stage-compensated spline data whereas the other, suffixed with “_orig” (*e. g.* ‘wt-1_spline_orig.txt’), contains the raw spline data without stage compensation and calibration.

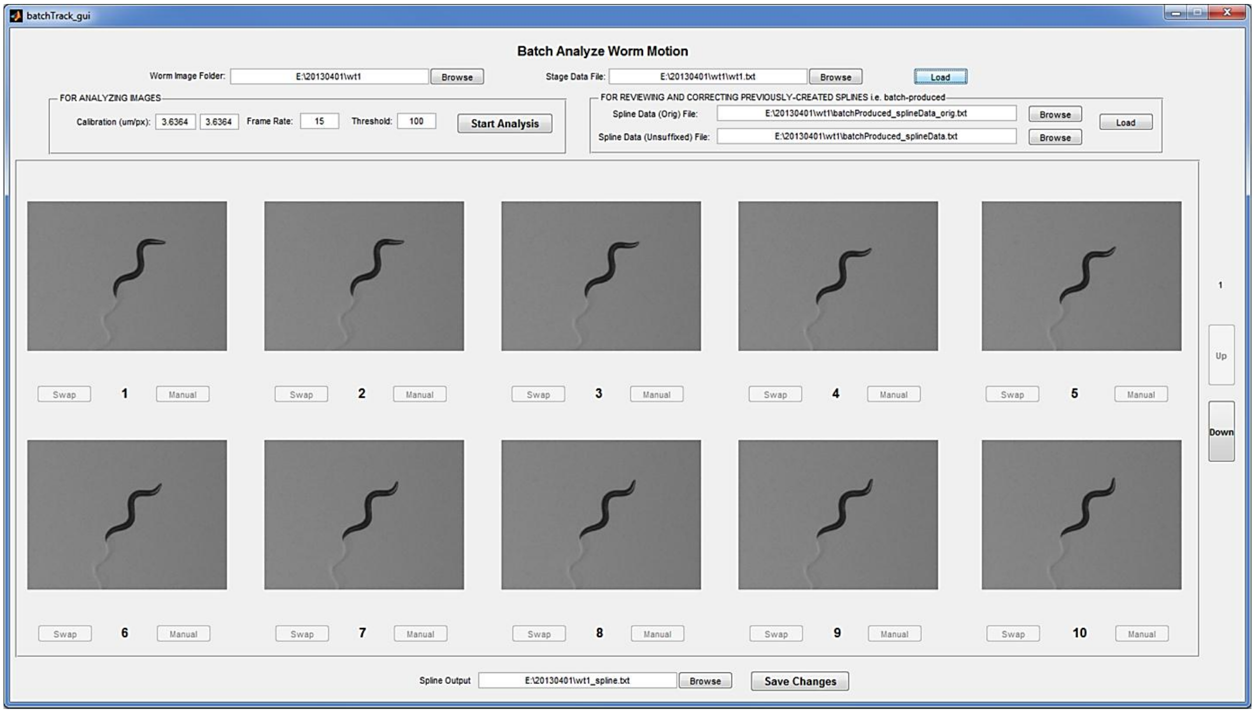

Figure S6. *Fit Spline* module prior to spline fitting.

The *Fit Spline* module may perform two functions: (1) generate splines for individual experiments, and (2) verify and correct splines generated by either the *Fit Spline* or *Batch Spline* module. In this section, we describe how to use *Fit Spline* to generate, inspect, and correct splines for individual experiments. A typical workflow is as follows:

1. Select a specific folder containing worm images. The corresponding stage file is automatically selected.
2. Press *Load* to observe the first 10 frames of the specific image folder appearing on the screen.
3. Enter the *x* and *y* calibration information and the frame rate used for the recording, and choose an appropriate threshold for binarization. The threshold value could be evaluated with the *Playback* module. *Track-A-Worm* has an internal mechanism to vary the threshold by as much as 20% from

the initial guess if a valid spline cannot be found using the selected threshold. Therefore, a spline can generally be fit even if the initial threshold was not optimal although the time to fit could be significantly longer due to repeated attempts. Since lighting conditions were generally similar for all the experiments of a batch, one threshold value may suits all the experiments.

4. Press *Start Analysis*. The initial detection window will appear with 13 markers along the spline and the candidate head indicated by an 'x' (**Figure S7**). You are prompted to either keep the head assignment or swap it with the tail. Since the head assignment in a preceding frame also serves as a reference for that in the following frame, it is important to verify the head assignment at the initial detection window.

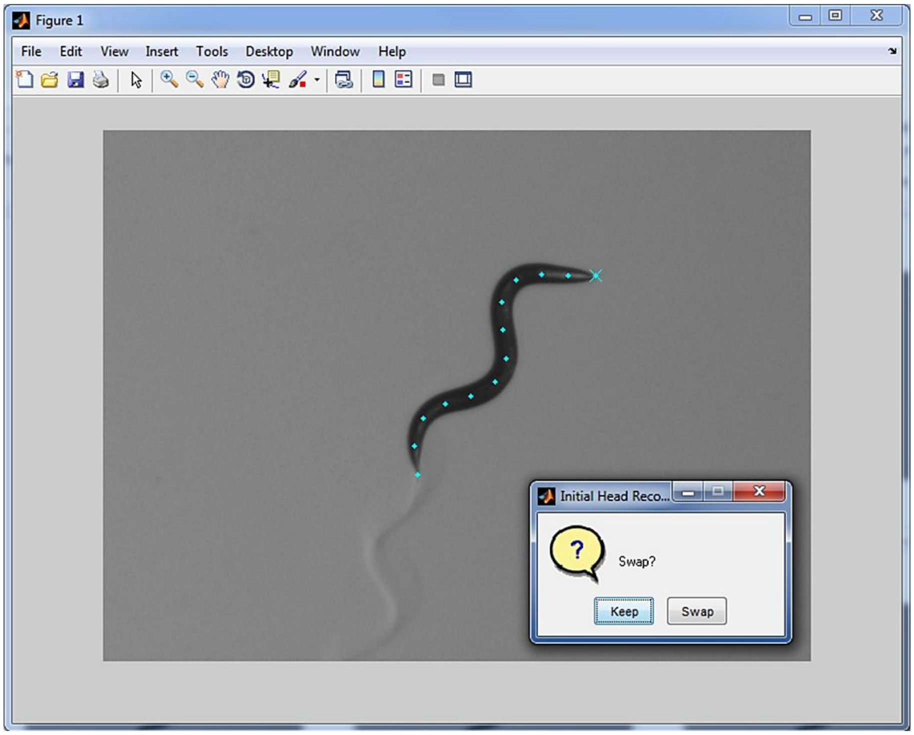

Figure S7. The initial detection window for verifying head assignment. This window appears at the beginning of spline fitting. Click *Keep* if the head assignment was correct but *Swap* if the head assignment was wrong. This window then disappears, which is followed by automatic spline fitting for the remaining frames.

5. After closing the initial detection window, the software will run on its own for a period of time. Each frame typically takes 1 second on a Core i7-920 PC but longer if the image is more challenging. During this period of time, the *Fit Spline* user interface appears to be stationary. The progress of spline fitting is displayed real-time in the Matlab command line window (**Figure S8**). When the analysis is finished, a spline is displayed over each worm image with the head marked by an '*x*' sign (**Figure S9)**.
6. The user should then inspect the spline fitting and head assignment results for all the frames of this recording. Five frames are advanced each time the *Down* button is clicked (**Figure S9**). Potential errors can be corrected as follows: **Incorrect head assignment** -- Click "Swap" to fix it. This action also causes head and tail swap for all the subsequent frames since head assignment in each subsequent frame was partially based on head position in the preceding frame during the automatic analysis. **Failed or poor spline fitting** -- Spline fitting for a specific frame may be fixed by first clicking the *Manual* button below it and then following the instructions in the legend of **Figure S10**.

7. After you have inspected spline fitting for all of the frames, click *Save Changes* to save the spline file. Note that the default folder and file name are automatically selected by *Track-A-Worm* although you may override it, which we do not recommend.

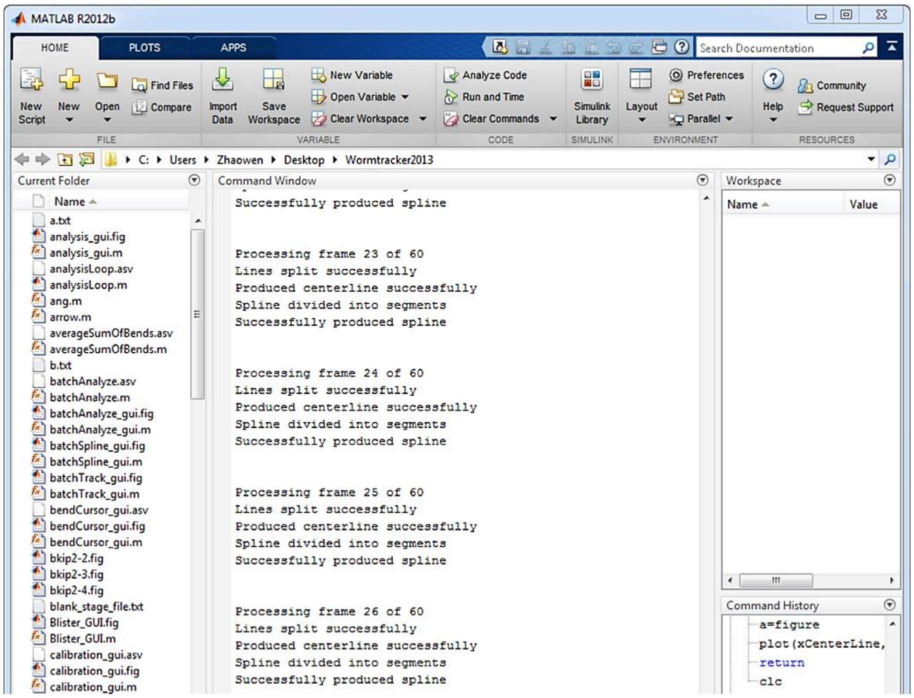

Figure S8. Matlab command line window.

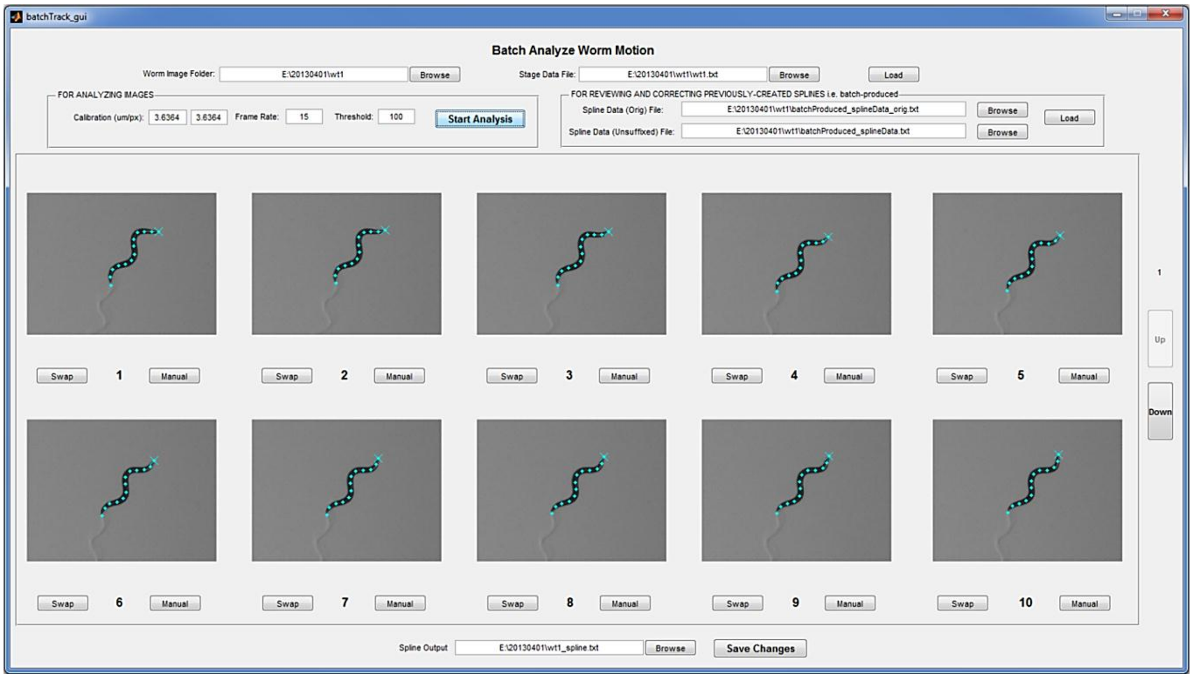

Figure S9. *Fit Spline* module after spline fitting. Each worm is superimposed by an ‘*x*’ sign marking the head location and 13 markers representing the deduced spline.

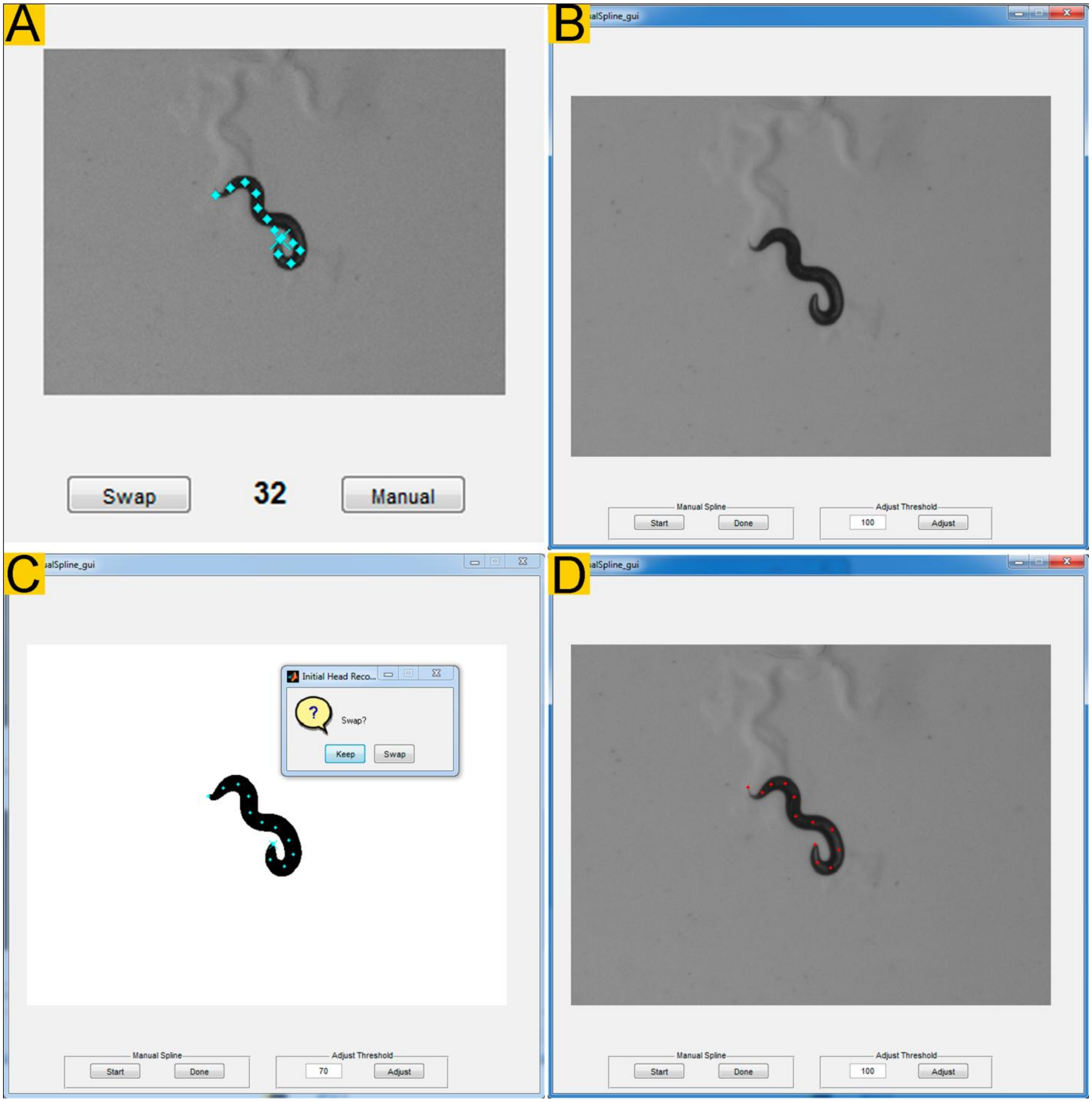

Figure S10. Correction of spline fitting. A. The snapshot of a frame from the *Analyze* module showing that the spline was poorly fit using a threshold value of 100. **B.** A new window pops out upon clicking the manual button in **A** showing the original image. **C.** After changing the threshold from 100 to 70 in the open box and clicking the *Adjust* button, 13 spline markers are placed appropriately over the worm with the head indicated by an '*x*'. If both the spline fitting and head assignment were satisfactory, click the *Keep* button in the small window. If the spline fitting was good but the head assignment was wrong, click the *Swap* button. If the spline was not successfully fit, try a new threshold or click the *Start* button to do manual spline fit. You will need to close and reopen the window shown in **B** to redo the analysis. **D.** Upon clicking the *Start* button in **B**, the user may provide guiding markers as shown by clicking along the midline of the worm 9 to 15 times (> 15 is fine) in the head to tail direction. Upon clicking the *Done* button, the head will be correctly identified, and 13 markers will be placed appropriately along the worm. This approach is more user-dependent but always works.

Batch Spline

The spline is fit through complicated computational processes. Automatic computation is the most time-consuming step in obtaining satisfactory spline fitting. In the *Fit Spline* module described above, the user needs to select the image folder, and inspect/correct the fitting results for one recording before moving onto the next one. Therefore, frequent user attention is needed to upload new recordings. The *Batch Spline* module (**Figure S11**) allows automatic spline fitting for many recordings in a batch. The user can then choose a convenient time to inspect and verify the results.

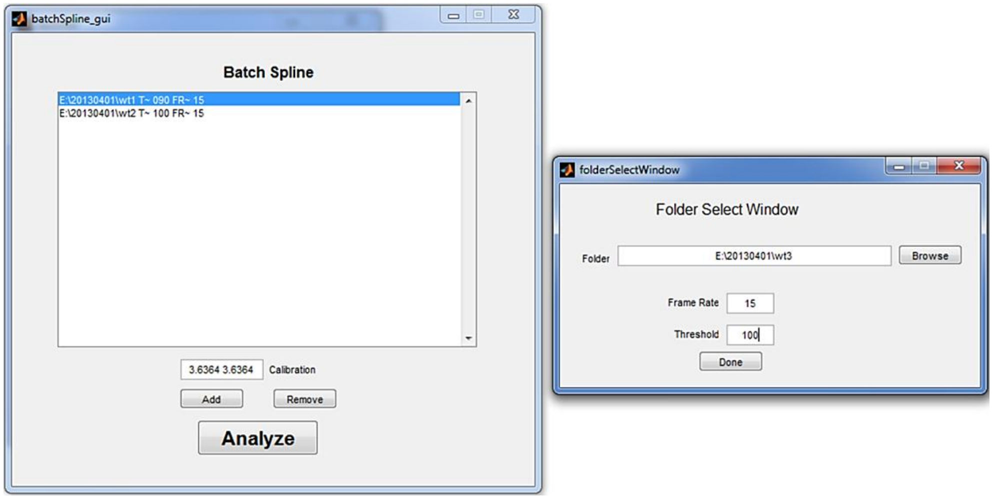

Figure S11. *Batch Spline* module. This module is used to perform automatic spline fitting for multiple recordings in a batch. The main user interface for this module is shown on the left. Clicking the *Add* button opens a new window (*right*), in which the user may select an image folder, enter the frame rate used for recording the images, and choose a threshold for binarization for a specific recording. Upon clicking the *Done* button, the selected image folder with the frame rate and threshold is added to the open box of the main user interface. Many recordings may be added by repeating these steps. If an undesired image folder was selected by accident, it can be removed by clicking the *Remove* button and entering a number matching its position in the list. Upon clicking the *Analyze* button, *Track-A-Worm* starts automatic spline fitting.

The results from the Batch Spline are saved in the original image folder as two files: ‘batchProduced_splineData.txt’ and ‘batchProduced_splineData_orig.txt’, which are with and without stage compensation/calibration, respectively. The next step is to inspect and correct the automatically generated spline data using the *Fit Spline* module by following these steps: (1) select a worm image folder using the *Browse* button on the top left, which results in automatic entry of the corresponding stage data file into the *Stage Data File* box; (2) click the *Load* button on the top right to observe the first 10 frames of the recording appearing on the screen without spline fitting; (3) click the *Load* button in the section titled *FOR REVIEWING AND CORRECTING PREVIOUSLY-CREATED SPLINES i. e. batch-produced* to observe the spline fitting results displayed over the worm images. The spline fitting files are automatically selected in this step although the user may override the automatic selection; (4) inspect and correct the spline fitting results as described earlier; and (5) save the verified spline data by clicking the *Save Changes* button at the bottom of the window. Again, the default folder and file name are entered automatically. Note that the default folder for the spline files is now one level higher than the image folder, which is the same as that for the spline files generated without using the *Batch Spline* module. In other words, no matter *Fit Spline* or *Batch Spline* was used for the initial automatic spline fitting, the final spine data are saved in the same folder and in the same format.

Analyze

The *Analyze* module (**Figure S12**) allows the user to perform all the analyses built into *Track-A-Worm*. It is typically used to perform quick analyses for single recordings, or to perform functions unique to this module, including plotting bend trace, bend frequency trace, and worm travel path. Another module known as *Batch Analyze* (described later) is used for simultaneous analyses of multiple worms.

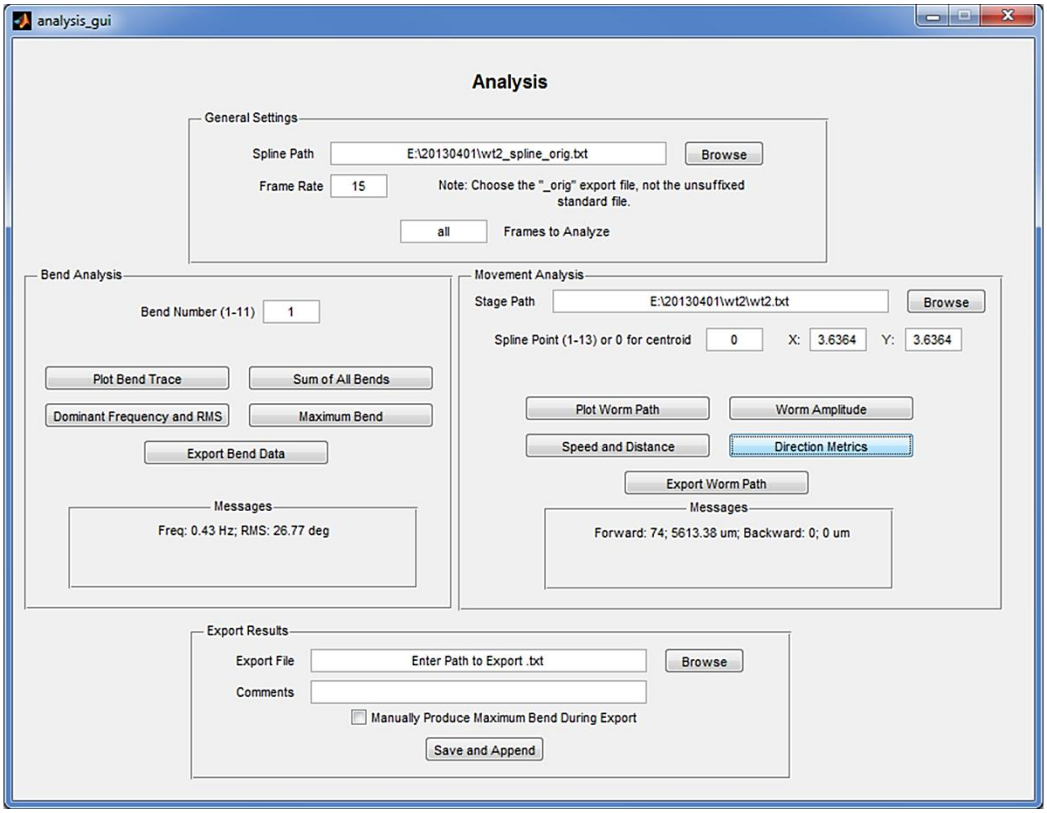

Figure S12. *Analyze* module user interface. The user first needs to select a spline file, enter the frame rate used for the recording, and select the range of frames for analysis under *General Settings*. As indicated in the *General Settings* box, spline files with the '_orig' suffix should be used. The frame range to analyze may be entered as either "all" or two numbers separated by a space (*e. g.* '31 80', which means frames 31 to 80).

Analytical features of this module are divided into two groups: *Bend Analysis* and *Movement Analysis*. In *Bend Analysis*, the user may specify a bend number to analyze. Any of the 11 bends formed by connecting the 13 markers along the spline may be selected. Several functions may be performed with *Bend Analysis*, including plotting the bend trace and bend frequency spectrum, quantifying the magnitude and frequency of maximum bends, quantifying the sum of all bends averaged over time, and reporting bending activities as RMS and maximum bend for any selected bend (1 to 11). In *Movement Analysis*, the user may reconstruct the worm travel path, report the worm amplitude, calculate the speed and distance of movements, and calculate the duration and distance of forward and backward movements. A corresponding stage file has to be selected to perform the movement analyses. The stage file is automatically selected unless it is not in the default folder. The various functionalities of the *Analyze* module are described below:

Plot Bend Trace

The bend trace is a plot of bend angle versus time for a selected bend. Its appearance gives the user some rough idea about bending properties. However, its most important use is to serve as the basis for detailed quantifications of bending properties as described later. A bend trace of the first bend (also known as “head bend”) of a wild-type worm is shown as an example (**Figure S13**).

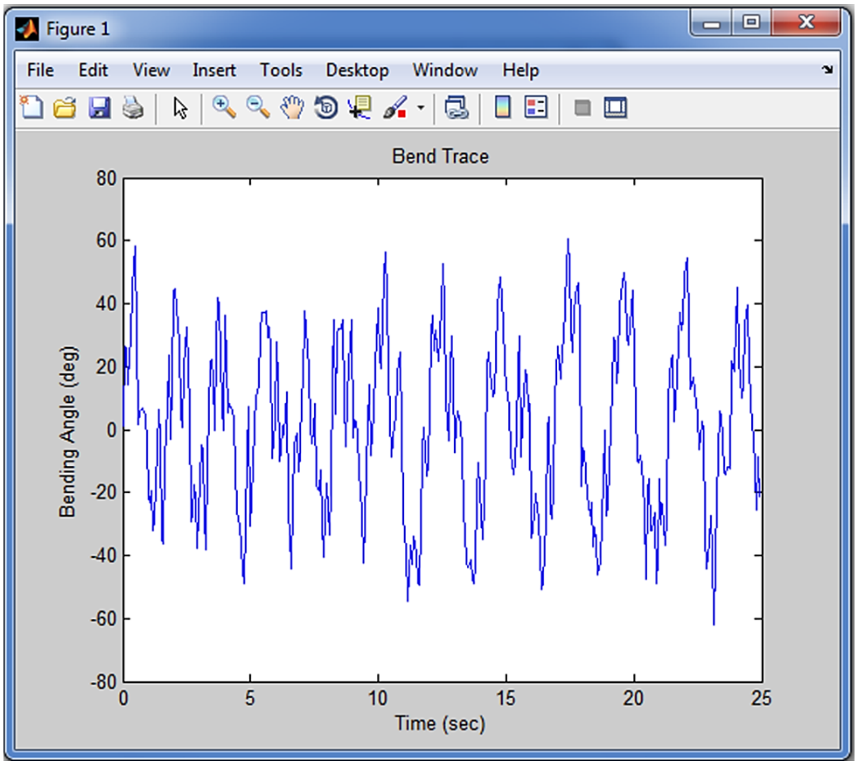

Figure S13. Bend trace of the first bend of a wild-type worm.

Dominant Frequency

A worm typically bends with one large dominant motion, with smaller random movements in between (**Figure S13**). Bend Frequency reports the frequency of the dominant bends in a bend trace. For example, a plot of the frequency spectrum (**Figure S14**) for the bend trace shown in **Figure S13** indicates that the dominant bend frequency is approximately 0.4 Hz.

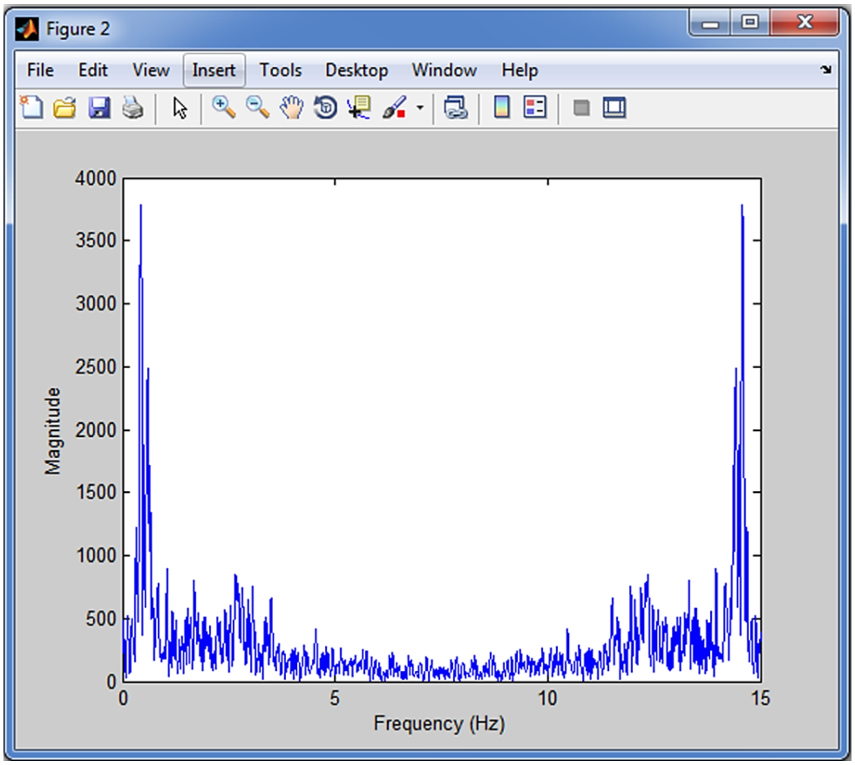

Figure S14. Bend frequency spectrum of the first bend of a wild-type worm. This graph is generated by Fourier transformation of the bend trace shown in **Figure S13**. The Fourier transformation always produces a mirror image. Thus, the user should disregard the second half of the graph. The main peak of this graph indicates that the dominant bending frequency is ~0.4 Hz.

Root Mean Square (RMS)

The bend frequency described above ignores the smaller and apparently random bending activities in the bend trace. However, changes of the smaller bends might be characteristic of some mutants. Therefore, it is important to include them in the quantification. *Track-A-Worm* uses RMS to measure all bending motions regardless of their amplitudes using the following formula:

$$RMS = \sqrt{\frac{\sum_i^n x_i^2}{n}}$$

where *n* is the total number of points and *i* refers to each individual point. While RMS is potentially more sensitive in detecting phenotypes of many mutants, it has limitations in distinguishing between high-frequency low-amplitude and low-frequency high-amplitude behavior.

Sum of All Bends

The *Sum of All Bends* is a useful metric for quantifying the bending behavior of an entire worm. It is simply the sum of all the 11 bends of a worm averaged over time. The *Sum of All Bends* metric uses the absolute values of the bends regardless of the directions of the bends.

Maximum Bend

It is often intuitive and useful to quantify the maximum “sweep” of a bend. Clicking the *Maximum Bend* button opens a bend trace of the selected bend, which allows the user to place markers at the most positive and negative points of each dominant bend by mouse-clicking while holding down the ALT key (**Figure S15**). *Maximum Bend* is calculated as the difference between the averages of the positive and negative values. This measure is only useful in worms that exhibit organized bending behavior. If the behavior is very chaotic without obvious extrema of movement, the RMS measurement may be more useful. Before quantifying the maximum bend, it is important to define a threshold. For example, the user may choose the most positive and negative peaks larger than 20 degrees alternatively.

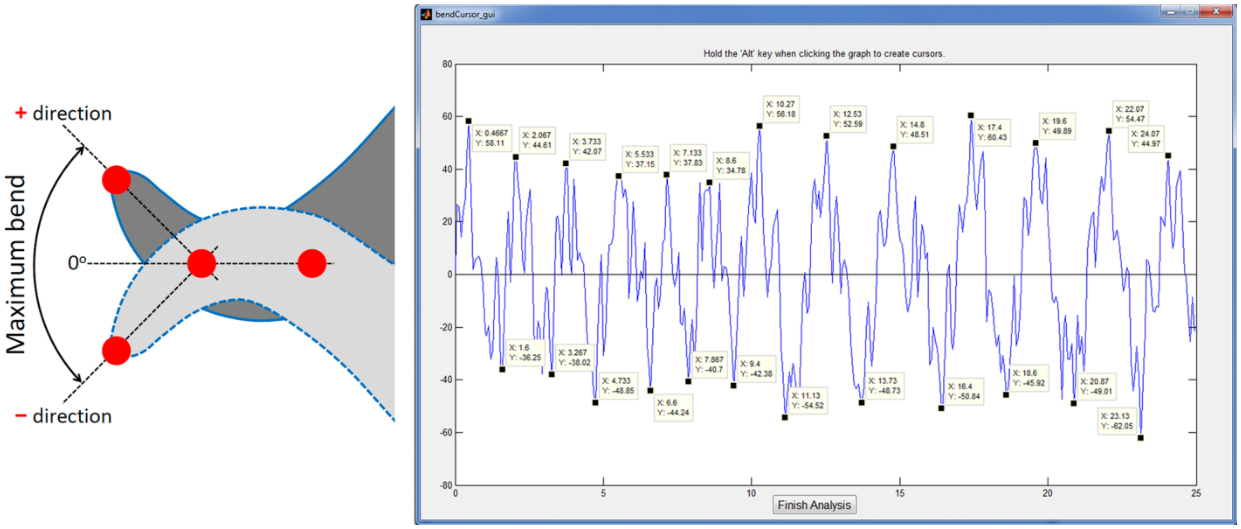

Figure S15. Quantification of the maximum bend. *Left,* A diagram using the first bend as an example to show the definition of the maximum bend. The red circles represent the first three markers along the spline. *Right,* Bend trace of the first bend of a wild-type worm (25 sec). Note that the bend trace is dominated by large-amplitude (alternating between approximately +50 to -50 degrees) bends but accompanied by smaller and apparently random oscillations. The *x* (time) and *y* (angle) values of the most positive and negative points of each dominant bend are displayed by mouse clicking at the bend trace. Upon clicking the *Finish Analysis* button, the difference between the averages of the most positive and negative values (94.61 degrees in this case) is automatically determined.

Worm Path

The traveling path of a worm can be reconstructed using this function. The plot may be based on the positions of either the centroid (by entering “0”) or any of the 13 markers along the worm spline (**Figure S16**). The worm path plot does not indicate the starting and ending points of the worm movements, which could be told from the txt file generated by clicking the *Export Worm Path* tab.

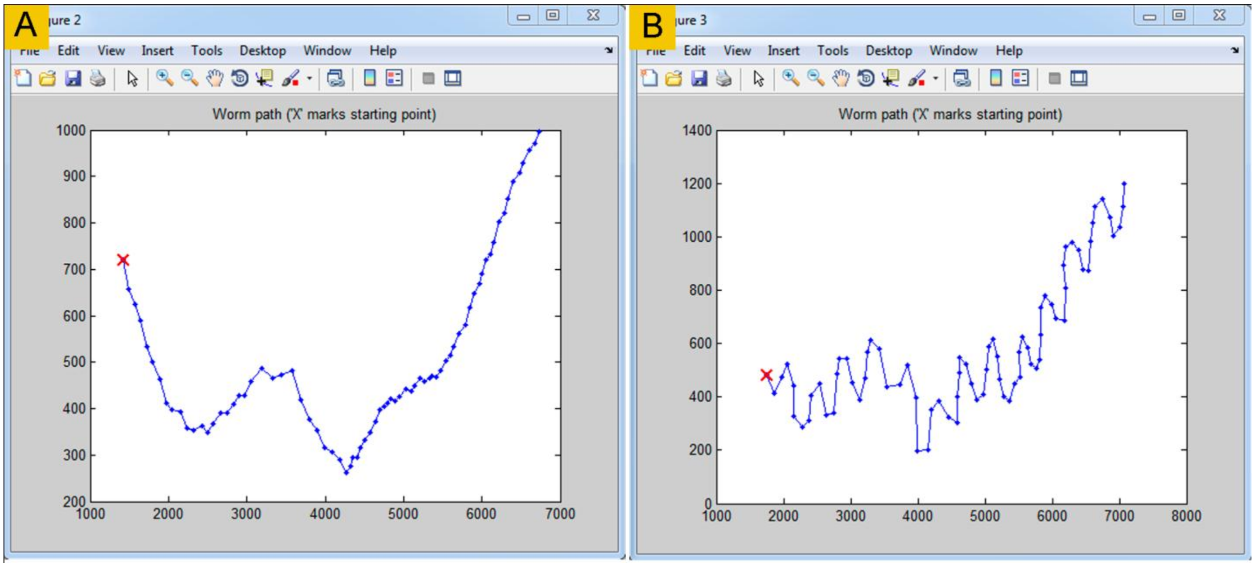

Figure S16. The travel path of a wild-type worm. A. Travel path of the centroid path. **B.** Travel path of marker #1. The *x* and *y* axis labels are in micrometers. The red “x” sign indicates the starting point of the worm path. The movement analyses were performed at a frame rate of 3 Hz.

Speed and Distance

The Speed function measures the average speed of a worm in µm/sec. The Distance function measures the total distance (µm) traveled by the worm (including both forward and backward movements) as well as the net distance travelled (the straight-line distance from the first position to the last position of the worm over the analyzed period). When accessing the Distance function through the *Analyze* (not the *Batch Analyze*), a plot of time versus absolute value speed will be produced (**Figure S17**).

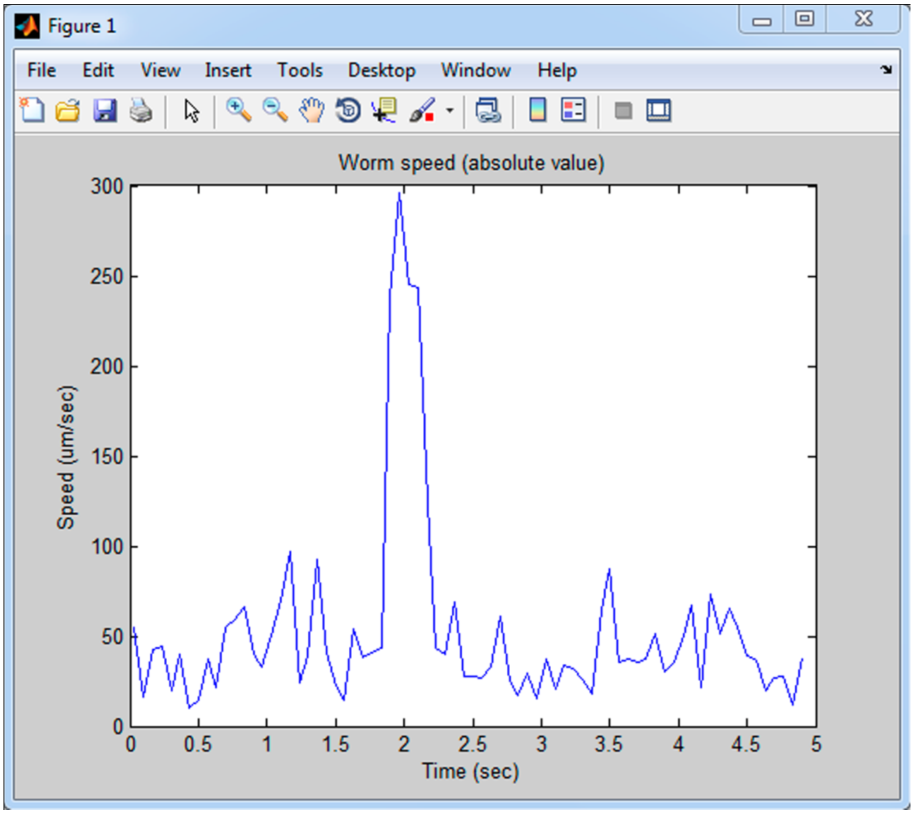

Figure S17. Plot of speed over time for an *unc-9(fc16)* mutant worm.

Direction Metrics

The direction function measures the number of frames in which a worm is moving forward and the number of frames it is moving backward. Directionality is determined by the velocity vector (a straight line from the centroid of the current frame to that of the next frame) and the head vector (a straight line from the current centroid to the current head tip position). The worm is considered to be moving forward if the projection of the head vector onto the velocity vector is positive, and vice versa (**Figure S18**). The direction function also outputs the total distances of forward and backward motion.

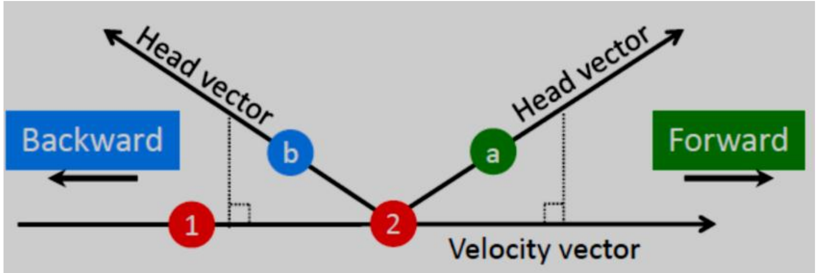

Figure S18. Determination of movement directions. The red circles represent the positions of the centroid at the current frame (#1) and the next frame (#2). The green circle labeled “a” and the blue circle labeled “b” show that the head vector is projected to the positive and negative sides of the velocity vector, respectively.

Worm Amplitude

Amplitude is another measure of worm curvature. A highly curved worm tends to have large amplitude, and vice versa. Amplitude is determined by first finding the velocity vector as described above. Then, a box is drawn in parallel with the velocity vector to enclose the widest points of the spline (not the actual worm body) (**Figure S19**). The width of the box is the worm amplitude. Because the amplitude could be affected by the length of the worm, *Track-A-Worm* also reports the ratio of the amplitude over the length of the worm (*A/L*).

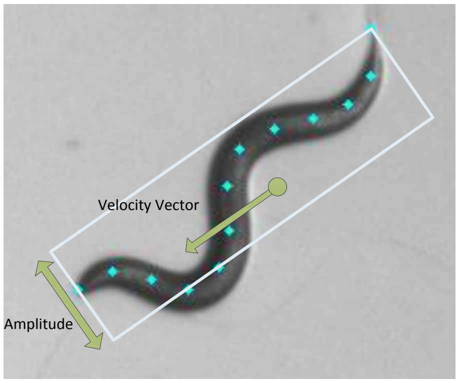

Figure S19. Determination of worm amplitude.

Results display and Export

Either a plot or the calculated result is displayed instantly upon clicking a specific functional tab in the *Analyze* module. A plot is displayed in a new window whereas calculated results are shown inside the *Messages* boxes. The *Analyze* module may also export the data for further analyses by clicking three different export buttons. The *Export Bend Data* button is used to export the angle values over time for any of the 11 bends. The *Export Worm Path* button is used to export the *x-y* coordinates over time for the active *Worm Path* plot. A worm path plot must first be produced to use this function. The *Export Results* button is used to export all the remaining results not included in the other two export functions. When using the *Export Results* function, the user may add comments, choose to include manually produced *Maximum Bend*, and append results from multiple experiments into the same file. The user should not modify the export file manually using Excel or any other application before the completion of appending new data to prevent corruption of the formatting. All the export files are tab-delimited txt files, which may be dragged into Excel to open (**Figure S20)**.

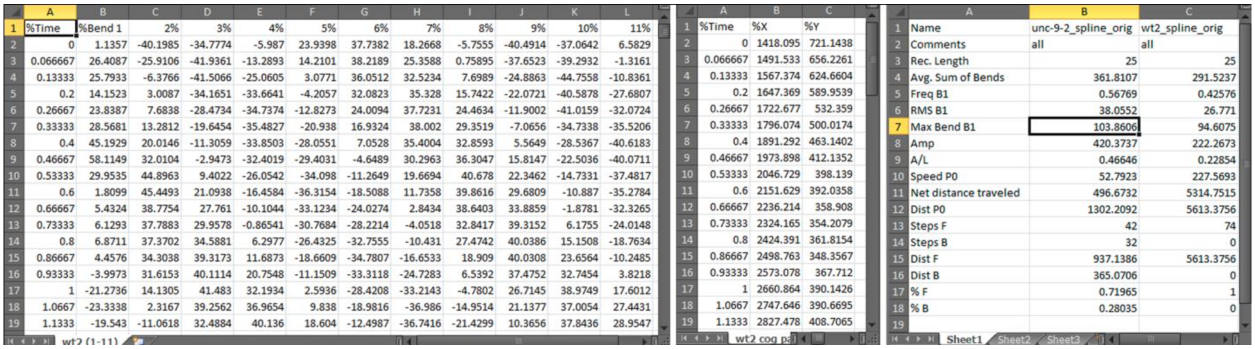

	A	B	C	D	E	F	G	H	I	J	K	L
1	%Time	%Bend 1	2%	3%	4%	5%	6%	7%	8%	9%	10%	11%
2	0	1.1357	-40.1985	-34.7774	-5.987	23.9398	37.7382	18.2668	-5.7555	-40.4914	-37.0642	6.5829
3	0.066667	26.4087	-25.9106	-41.9361	-13.2893	14.2101	38.2189	25.3588	0.75895	-37.6523	-39.2932	-1.3161
4	0.13333	25.7933	-6.3766	-41.5066	-25.0605	3.0771	36.0512	32.5234	7.6989	-24.8863	-44.7558	-10.8361
5	0.2	14.1523	3.0087	-34.1651	-33.6641	-4.2057	32.0823	35.328	15.7422	-22.0721	-40.5878	-27.6807
6	0.26667	23.8387	7.6838	-28.4734	-34.7374	-12.8273	24.0094	37.7231	24.4634	-11.9002	-41.0159	-32.0724
7	0.33333	28.5681	13.2812	-19.6454	-35.4827	-20.938	16.9324	38.002	29.3519	-7.0656	-34.7338	-35.5206
8	0.4	45.1929	20.0146	-11.3059	-33.8503	-28.0551	7.0528	35.4004	32.8593	5.5649	-28.5367	-40.6183
9	0.46667	58.1149	32.0104	-2.9473	-32.4019	-29.4031	-4.6489	30.2963	36.3047	15.8147	-22.5036	-40.0711
10	0.53333	29.9535	44.8963	9.4022	-26.0542	-34.098	-11.2649	19.6694	40.678	22.3462	-14.7331	-37.4817
11	0.6	1.8099	45.4493	21.0938	-16.4584	-36.3154	-18.5088	11.7358	39.8616	29.6809	-10.887	-35.2784
12	0.66667	5.4324	38.7754	27.761	-10.1044	-33.1234	-24.0274	2.8434	38.6403	33.8859	-1.8781	-32.3265
13	0.73333	6.1293	37.7883	29.9578	-0.86541	-30.7684	-28.2214	-4.0518	32.8417	39.3152	6.1755	-24.0148
14	0.8	6.8711	37.3702	34.5881	6.2977	-26.4325	-32.7555	-10.431	27.4742	40.0386	15.1508	-18.7634
15	0.86667	4.4576	34.3038	39.3173	11.6873	-18.6609	-34.7807	-16.6533	18.909	40.0308	23.6564	-10.2485
16	0.93333	-3.9973	31.6153	40.1114	20.7548	-11.1509	-33.3118	-24.7283	6.5392	37.4752	32.7454	3.8218
17	1	-21.2736	14.1305	41.483	32.1934	2.5936	-28.4208	-33.2143	-4.7802	26.7145	38.9749	17.6012
18	1.0667	-23.3338	2.3167	39.2562	36.9654	9.838	-18.9816	-36.986	-14.9514	21.1377	37.0054	27.4431
19	1.1333	-19.543	-11.0618	32.4884	40.136	18.604	-12.4987	-36.7416	-21.4299	10.3656	37.8436	28.9547

wt2 (1-11)

	A	B	C
1	%Time	%X	%Y
2	0	1418.095	721.1438
3	0.066667	1491.533	656.2261
4	0.13333	1567.374	624.6604
5	0.2	1647.369	589.9539
6	0.26667	1722.677	532.359
7	0.33333	1796.074	500.0174
8	0.4	1891.292	463.1402
9	0.46667	1973.898	412.1352
10	0.53333	2046.729	398.139
11	0.6	2151.629	392.0358
12	0.66667	2236.214	358.908
13	0.73333	2324.165	354.2079
14	0.8	2424.391	361.8154
15	0.86667	2498.763	348.3567
16	0.93333	2573.078	367.712
17	1	2660.864	390.1426
18	1.0667	2747.646	390.6695
19	1.1333	2827.478	408.7065

wt2 cog pa

	A	B	C
1	Name	unc-9-2_spline_orig	wt2_spline_orig
2	Comments	all	all
3	Rec. Length	25	25
4	Avg. Sum of Bends	361.8107	291.5237
5	Freq B1	0.56769	0.42576
6	RMS B1	38.0552	26.771
7	Max Bend B1	103.8606	94.6075
8	Amp	420.3737	222.2673
9	A/L	0.46646	0.22854
10	Speed P0	52.7923	227.5693
11	Net distance traveled	496.6732	5314.7515
12	Dist P0	1302.2092	5613.3756
13	Steps F	42	74
14	Steps B	32	0
15	Dist F	937.1386	5613.3756
16	Dist B	365.0706	0
17	% F	0.71965	1
18	% B	0.28035	0
19			

Sheet1 Sheet2 Sheet3

Figure S20. Data exported by the *Analyze* module. The *Analyze* module may export three different data sets including Bend Data (*left*), Worm Path (*middle*), and Results (*right*) by clicking the corresponding export tabs. In the Bend Data spreadsheet, the first column indicates time (sec) while the remaining columns show the angles of the 11 different bends at each time point. In the Worm Path spreadsheet, the first column indicates time (sec) while the remaining two columns indicating the x and y coordinates of the centroid at each time point. The Results file could contain data from multiple recordings (show are data from two recordings). In the Results spreadsheet, the first column shows various row labels, including the point number (0 or centroid in this case), the bend number (bend 1 in this case), comments (anything from the user), recording length (sec), average sum of all bends (degrees), frequency of dominant bends (Hz), mean amplitude of maximum bends (degrees), average amplitude (µm), ratio of amplitude over worm length (A/L), average speed (µm/sec), total distance traveled (µm), the number of forward steps, the number of backward steps, the total distance of forward movement, and the total distance of backward movement. The remaining two columns show the data from one wild-type worm and one *unc-9(fc16)* mutant worm.

Batch Analysis

The *Batch Analyze* module (**Figure S21**) may perform all the quantifications described for the *Analyze* module. It is better suited to analyzing large sets of data. Recordings are added via a small pop-up interface (**Figure S21**).

Analyses are chosen via checkbox. Bend Numbers are chosen using standard Matlab syntax. You can choose individual bends using the format ‘1 3 5’, indicating discrete points. You can choose entire ranges of bends using the format ‘1:5’, indicating a series of connected points. Remember that there are a total of 11 bends for a worm spline. Point numbers are also chosen using the same syntax. However, the choice ‘0’ is used in this context to select the centroid. For example, you could select the centroid, head tip, and tail by typing ‘0 1 13’ into the box. Calibration is entered simply by entering the *x* and *y* values separated by a space. When all settings have been selected correctly, simply choose the save path for the txt file, and click *Analyze and Export*.

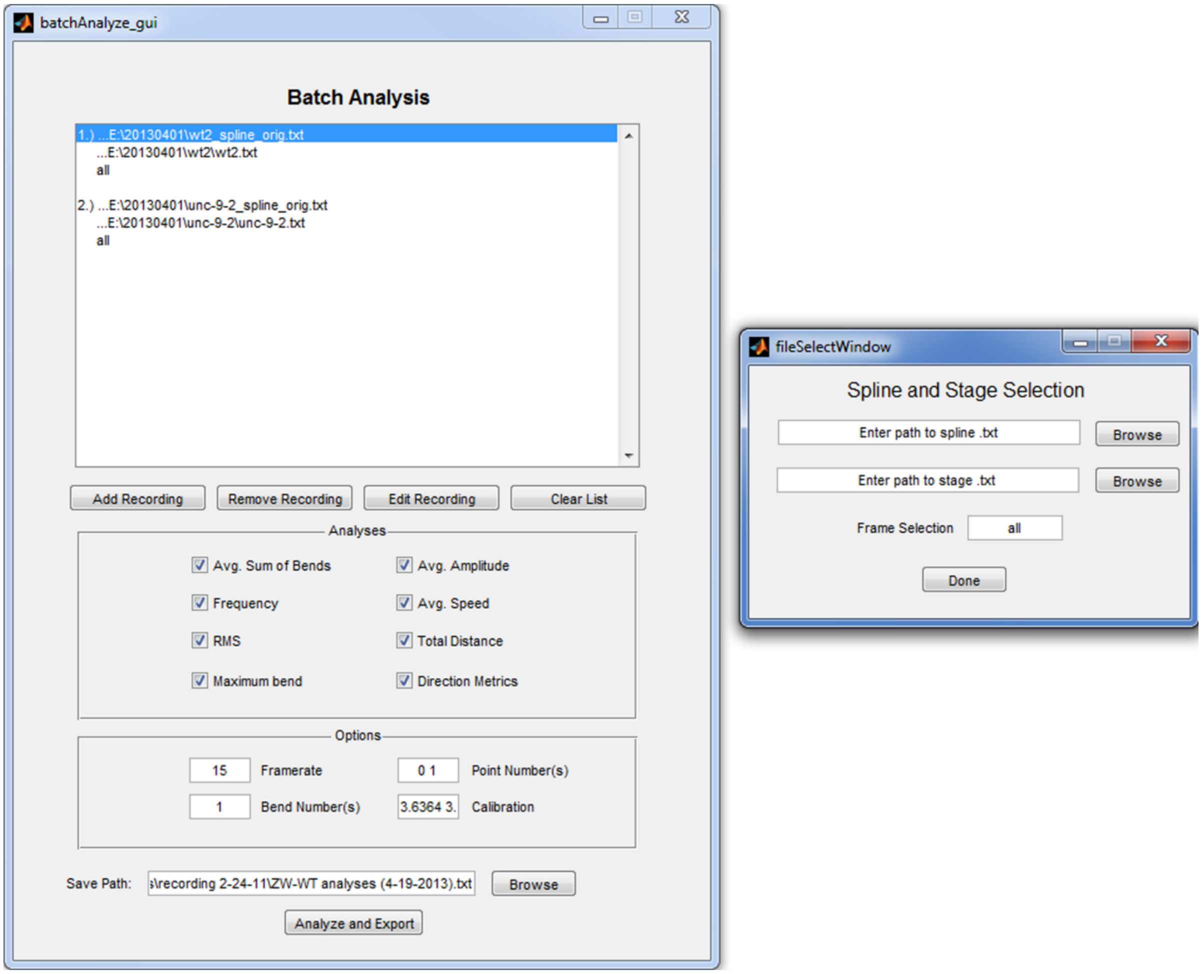

Figure S21. *Batch Analyze* module. Multiple recordings may be analyzed simultaneously using this module. The user may select a list of recordings by clicking the *Add Recording* button in the main user interface (*left*), pick the parameters for quantification, and choose the *Bend Number(s)* for bend analyses and *Point Number(s)* for movement analyses. The small window (*right*) pops out upon clicking the *Add Recording* button in the main user interface to allow the user select specific spline files (suffixed with “_orig”). When a specific spline file is selected, the corresponding stage file is automatically selected. Frame selection is performed using the same syntax described earlier (*e. g.* enter “1:50” to select frames 1-50 or “all” to select the entire recording).
